# Viable but non-cultivable state in oral microbiota: a critical review of an underexplored microbial survival strategy

**DOI:** 10.3389/fcimb.2025.1533768

**Published:** 2025-03-18

**Authors:** Marzie Mahdizade Ari, Konstantin Johannes Scholz, Fabian Cieplik, Ali Al-Ahmad

**Affiliations:** ^1^ Department of Microbiology, School of Medicine, Iran University of Medical Sciences, Tehran, Iran; ^2^ Department of Operative Dentistry and Periodontology, Center for Dental Medicine, Faculty of Medicine and Medical Center, University of Freiburg, Freiburg im Breisgau, Germany

**Keywords:** dormancy, viable but non-cultivable, VBNC, persister cells, resuscitation, virulence, infectious diseases, detection methods

## Abstract

The viable but non-cultivable (VBNC) state and persister cells, two dormancy phenomena in bacteria, differ in various aspects. The entry of bacteria into the VBNC state as a survival strategy under stressful conditions has gained increasing attention in recent years, largely due to the higher tolerance of VBNC cells to antibiotics and antimicrobials resulting from their low metabolic activity. The oral cavity favors biofilm growth in dental hard tissues, resulting in tooth decay and periodontitis. Despite advances in VBNC state detection in the food industry and environment, the entry capability of oral bacteria into the VBNC state remains poorly documented. Furthermore, the VBNC state has recently been observed in oral pathogens, including *Porphyromonas gingivalis*, which shows potential relevance in chronic systemic infections, *Enterococcus faecalis*, an important taxon in endodontic infections, and *Helicobacter pylori*, which exhibits transient presence in the oral cavity. Further research could create opportunities to develop novel therapeutic strategies to control oral pathogens. The inability of conventional culture-based methods to identify VBNC bacteria and the metabolic reactivation of dormant cells to restore susceptibility to therapies highlights a notable gap in anti-VBNC state strategies. The lack of targeted approaches tested for efficacy against VBNC bacteria underscores the need to develop novel detection methods. This review discusses the VBNC state, its importance in public health, and diagnostic techniques, with a special focus on the VBNC state in oral bacteria.

## 1 Introduction

The human body harbors approximately 3.8 × 10^13^ bacteria, a number comparable to the estimated 3.0 × 10^13^ human cells (Sender et al., 2016). Understanding microbial communities, their individual members, and interactions with host cells is crucial for clinical research and healthcare. However, this understanding is heavily influenced by the analytical and detection methods used, both clinically and *in vitro*. Many bacteria have long eluded examinations due to their inability to be cultivated and studied in traditional laboratory settings (Wade, 2002). Advances in nucleotide sequencing technologies for DNA and RNA analyses have revolutionized the study of non-cultivable microbial strains, uncovering that these previously inaccessible microbes represent the vast majority of microbial life (Kumar et al., 2021). In clinical settings, bacteria can adopt states that enable them to remain viable and pathogenic within the host organism while becoming non-cultivable in laboratory conditions (Stewart, 2012). These phenomena pose marked challenges for the accurate diagnosis and development of effective antibacterial strategies (Fleischmann et al., 2021). The bacterial stress response is closely linked to a markedly reduced growth rate and susceptibility to antibiotics, contributing to increased dormancy in biofilms. Certain bacterial cells evade antibiotic effects by entering a dormant state, a key adaptive strategy that allows survival under adverse conditions. Dormancy enables bacteria to maintain low metabolic activity and minimal or no growth while retaining the ability to resume division when conditions become favorable (Wood et al., 2013). Bacterial dormancy states, including the viable but non-cultivable (VBNC) state and bacterial persisters, represent highly stress-tolerant physiological adaptations that have been extensively studied (Ayrapetyan et al., 2018). Environmental stresses considerably enhance bacterial tolerance within biofilms compared to planktonic cells. This stress-induced tolerance creates heterogeneity in the population, leading to the emergence of antibiotic-tolerant cells, including VBNC and persister cells. These cells markedly contribute to antimicrobial treatment failures and infection persistence (Stewart and Franklin, 2008; Nguyen et al., 2011). While persister and VBNC state cells are stress-induced survival subpopulations that are alive, metabolically active, but not actively replicating (Bao et al., 2023), they differ in key ways. Few studies have explored these differences, largely due to the difficulty in distinguishing between the VBNC state and persister states under coexistence. Ayrapetyan et al. hypothesized that VBNC cells and persisters form part of a dormancy continuum, where active cells under stress transition into persisters, which may further develop into VBNC state cells (Ayrapetyan et al., 2015a). To test this, they compared the ability of persister and log-phase *Vibrio vulnificus* cells to enter the VBNC state (Ayrapetyan et al., 2015b). Log-phase cells took 7–10 days at 4°C to enter the VBNC state, with only 1%–10% resuscitating upon temperature increase. In contrast, persister cells isolated through antibiotic treatment transitioned into the VBNC state faster (4–5 days). This suggests that persisters are more efficient at becoming VBNC, potentially due to stress from prior antibiotic exposure or the lower initial cell numbers after treatment. This hypothesis corroborates Oerman and Brynildsen’s findings, which showed that an abundant aggregation of VBNC state cells was accompanied by the presence of a small number of persister cells (Orman and Brynildsen, 2013). Therefore, bacterial pathogen dormancy enables resistance to antimicrobial strategies and environmental stress, potentially leading to reinfection or persistent infection in clinical settings (Li et al., 2014).

A biofilm is a structured microbial community encased in a self-produced extracellular matrix that protects microbes against adverse conditions, such as pH fluctuations, nutrient deprivation, immune defenses, and antimicrobial agents (Karygianni et al., 2020; Jakubovics et al., 2021). In the oral cavity, nutrient-rich environments allow bacteria to colonize surfaces such as teeth, soft tissues, dental implants, and restorative materials, with salivary pellicle proteins facilitating initial adhesion, microbial growth, and biofilm formation (Costa et al., 2023). These biofilms contribute to dental caries, periodontal diseases, and tooth loss (Ray and Pattnaik, 2024). Therefore, they are challenging to treat due to enhanced co-aggregation, microbial interactions, and resistance to antimicrobial agents and host defense (Hajishengallis et al., 2011; Bowen et al., 2018). The ability of oral bacteria to enter the VBNC state opens a new dimension to the persistence and resilience of oral biofilms, further complicating their management and contributing to chronic oral infections. While the ability of oral bacteria to enter the VBNC state remains underexplored, recent studies confirm that pathogens like *P. gingivalis*, *E. faecalis*, and *H. pylori* can adopt this state, revealing an additional survival mechanism. This review article aims to comprehensively analyze the existing literature on the VBNC state of bacteria, with a focus on oral pathogens such as *P. gingivalis*, *E. faecalis*, and transient *H. pylori*. The study of VBNC bacteria is crucial, as they can evade conventional detection and antimicrobial treatments by entering a dormant state. This review explores the mechanisms of entering and resuscitating from the VBNC state, clinical implications, conventional and novel detection methods, and potential therapeutic strategies. Understanding the role of the VBNC state in oral pathogens will contribute to developing novel approaches for managing oral diseases and improving patient outcomes.

## 2 VBNC state in bacteria

**Figure 1:**
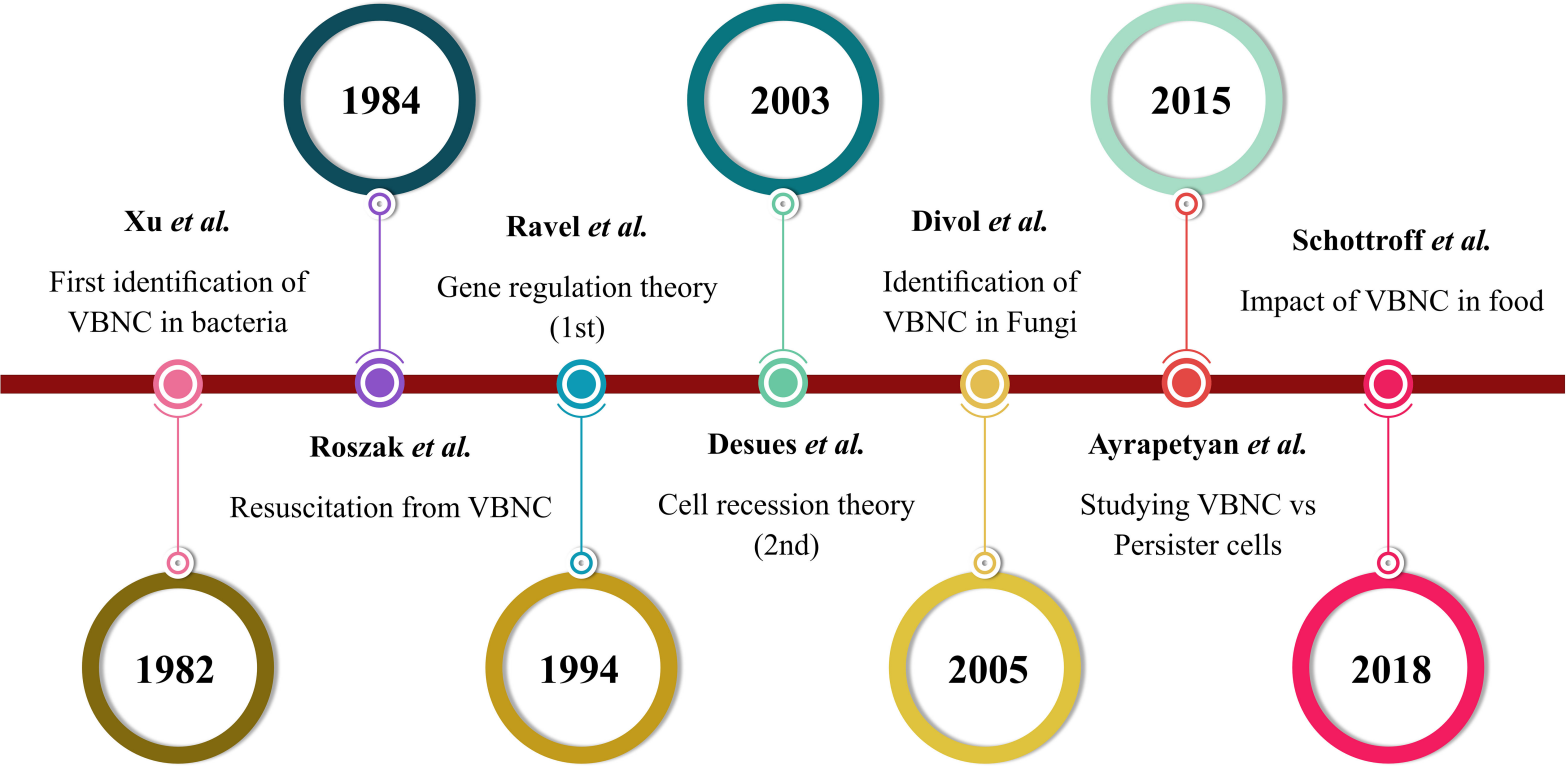
VBNC state timeline based on the literature.

Many bacteria readily enter a temporal state of dormancy, known as the VBNC state, to manage environmental stress. The concept of a critical population of non-cultivable cells was first described in 1982 by Xu and Colwell et al (Xu et al., 1982). As the timeline shows in **Figure 1**, the term “viable but non-cultivable” (VBNC) was introduced in 1984 (Colwell et al., 1985). Then, the key theories on VBNC state formation emerged in 1994 (Ravel et al., 1994) and 2003 (Desnues et al., 2003), and Divol et al. identified the VBNC state in fungal species for the first time (Divol and Lonvaud-Funel, 2005). Research continues to advance the understanding of VBNC states across food, environmental, and clinical settings, especially VBNC cells from persister cells (Ayrapetyan et al., 2015b; Schottroff et al., 2018). The VBNC state in bacteria is typically defined by two key features: decelerated growth rate and reduced metabolic activity (Wagley et al., 2021). However, this dormant condition is not permanent, and when the environmental stress that induces the VBNC state is removed, these bacteria populations can regain their full metabolic capacity (Lennon and Jones, 2011; Ayrapetyan et al., 2014). VBNC cells are also known as “active but non-cultivable cells” (ABNC), “conditionally viable environmental cells” (CVEC), “nongrowing but metabolically active” (NGMA) (Manina and McKinney, 2013), and “viable but apparently non-cultivable” (VPNC) (Nelson et al., 2008; Kamruzzaman et al., 2010; Deng et al., 2015; Dolezalova and Lukes, 2015). Currently, VBNC state cells are defined as non-cultivable microbial cells with the potential to revert to a growth state and proliferate in a growth medium. Despite reduced metabolic activity, they retain membrane integrity, and translational dynamics remain active (Ayrapetyan et al., 2014; Ayrapetyan et al., 2015b; Ayrapetyan and Oliver, 2016). In addition to lower metabolic activity, VBNC state cells undergo many changes in proteins, fatty acid levels, and peptidoglycan structure. For example, the *E. faecalis* VBNC state showed higher levels of peptidoglycan crosslinking than cultivable *E. faecalis* (Signoretto et al., 2000). In addition, alterations in outer membrane protein (Omp) levels have been observed in *E. coli* during the VBNC state (Muela et al., 2008), with Omp W showing a marked increase in this state (Asakura et al., 2008). *V. vulnificus* exhibited increased levels and structural changes in unsaturated fatty acids upon transitioning to the VBNC state, including a notable shift toward fatty acids with fewer than 16 carbon atoms and elevated levels of octadecanoic and hexadecanoic acids (Day and Oliver, 2004). Comparing VBNC cells to their cultivable counterparts reveals varying gene expression profiles. For instance, *Vibrio cholerae* was found to upregulate genes associated with regulatory functions, cellular processes, energy metabolism, transport, and binding activity more than fivefold (Asakura et al., 2007). In addition, increased expression of VBNC state genes associated with metabolism, cell cycle regulation, regulatory processes, and binding ability was observed. However, Cheng et al.’s investigation showed that, compared with cultivable cells, VBNC state cells had downregulated transcription levels of genes linked to adhesion, invasion, motility, and tolerance to toxic environmental stress (Guo et al., 2023). The strong capacity for resilience to varying stress situations is demonstrated by VBNC state cells owing to the aforementioned physiological and regulatory alterations (Progulske-Fox et al., 2022). Identifying dormant cells is crucial for public health, as their slow-growing nature prevents detection by culture-based strategies while allowing them to maintain virulence during dormancy (Baffone et al., 2003; Baffone et al., 2006; Oliver, 2010).

## 3 VBNC state formation theories

Gene regulation (Ravel et al., 1994) and cell recession (Desnues et al., 2003) are two primary theories proposed to explain the mechanism underlying bacterial VBNC state development. The latter suggests that adverse environmental conditions, such as oxidative stress, can prevent bacterial growth, leading to VBNC state formation (Marinelli et al., 2017), whereas the former suggests that bacteria incapable of producing spores utilize genetic regulatory mechanisms to enter the VBNC state as a survival strategy. This theory has led to the identification of various genes and proteins involved in initiating and controlling the VBNC state, highlighting its genetic basis (Roszak and Colwell, 1987; Zhao et al., 2017). Ravel et al. published the first report on the genetic control of VBNC formation in *V. cholerae*, describing it as a genetically programmed survival mechanism triggered by adverse conditions, such as low temperatures and reduced nutrient availability. Using transposon mutagenesis, over 2,500 mutants were screened, and mutant JR09H1 showed a faster entry into the VBNC state at 4°C, becoming non-cultivable after 13 days, compared to the wild type, which remained cultivable for 27 days. This research advanced the understanding of the genetic mechanisms controlling the VBNC state (Oliver et al., 2005).

So far, VBNC states have been described in *Aeromonas hydrophila*, *Aeromonas salmonicida*, *Agrobacterium tumefaciens*, *Burkholderia cepacia* and *Burkholderia pseudomallei*, *Campylobacter coli*, *Campylobacter Jejuni*, *Campylobacter Lari*, *Cytophaga allerginae*, *Enterobacter aerogenes*, *Enterobacter cloacae*), *Enterococcus faecalis*, *Enterococcus hirae*, *Enterococcus faecium*), *Erwinia amylovora*, *Escherichia coli* (EHEC), *Francisella tularensis*, *Helicobacter pylori*, *Klebsiella aerogenes*, *Klebsiella pneumoniae*, *Klebsiella planticola*, *Legionella pneumophila*, *Listeria monocytogenes*, *Micrococcus luteus*, *Mycobacterium tuberculosis*, *Mycobacterium smegmatis*, *Pasteurella piscicida*, *Pseudomonas aeruginosa*, *Pseudomonas syringae*, *Pseudomonas putida*), *Rhizobium leguminosarum*, *Rhizobium meliloti*, *Salmonella enterica*, *Ssalmonella Typhi*, *Salmonella Typhimurium*, *Serratia marcescens*, *Shigella dysenteriae*, *Shigella flexneri*, *Shigella sonnei*), *Vibrio alginolyticus*, *Vibrio anguillarum*, *Vibrio campbellii*, *V. cholerae*, *Vibrio harveyi*, *Vibrio mimicus*, *Vibrio parahaemolyticus*, *Vibrio shiloi*, *Vibrio vulnificus*), *Xanthomonas* spp. and *Yersinia* spp (Oliver, 2005; Oliver, 2010; Pazos-Rojas et al., 2019; Pazos-Rojas et al., 2023).

The VBNC state represents an adaptive survival strategy, typically triggered by stress factors such as temperature changes, antibiotic pressure, starvation, exposure to heavy metals, variations in oxygen concentrations, osmotic stress, deviations from the ideal pH range (such as during the stationary growth phase), lysosome activity, and ATP availability, which determines whether the organism enters the VBNC state. These factors allow bacteria to survive in harsh, nutrient-poor conditions where they are no longer detectable (Cunningham et al., 2009; del Campo et al., 2009; Li et al., 2014; Kortebi et al., 2017; İzgördü Ö et al., 2022).

A transcriptome analysis by Yang et al. revealed that genes involved in ATP production were significantly downregulated in cells exposed to stress treatments. ATP measurements further confirmed a marked decrease in ATP levels following exposure to heat, acidity, and long-term pre-adaptation cultivation. These results suggest that the environmental stress-induced reduction in ATP levels plays a key role in triggering VBNC state formation in *E. coli* (Yang et al., 2023). Similarly, *Yersinia pestis* became non-cultivable after 21 days in low-temperature tap water, exhibiting a significant reduction by 6 log_10_ steps in cultivable cells. Chlorination can also trigger VBNC state induction in *E. coli* and *Salmonella typhimurium* (Oliver et al., 2005; Pawlowski et al., 2011). Culture supernatants from the amoebae *Hartmannella vermiformis* and *Acanthamoeba polyphaga* revealed that *H. vermiformis* significantly inhibited the growth of *Legionella pneumophila*, reducing its cultivability by 3 log_10_ steps colony-forming units (CFU/mL) after 3 days of exposure. The extracellular polar signaling inhibitory molecules in *H. vermiformis* were primarily found in the <5 kDa fraction and appeared to be polar (Buse et al., 2013).

## 4 Parameters for assessing bacteria in the VBNC state and distinguishing from persister cells

Metabolic activity levels can be used to distinguish VBNC cells from other dormant or dead cells. VBNC cells retain measurable metabolic activity, whereas persister cells display a minimal level and often remain undetectable (Oliver, 2010; Fakruddin et al., 2013). These cells are typically isolated by exposing a growing bacterial culture to a lethal dose of antibiotics (Ayrapetyan et al., 2018). Chronic wound infections are often associated with biofilms, which serve as both a physical barrier and a protective niche for bacterial populations including persister cells. These cells contribute to antibiotic tolerance by limiting antibiotic penetration into biofilms, along with reduced metabolic activity and slower bacterial growth (Poole, 2012). Golmoradi Zadeh et al. identified the type II toxin-antitoxin (TA) system, RelBE/RelE, in *P. aeruginosa* and demonstrated its role in influencing persister cell formation within biofilms (Zadeh et al., 2022). The study showed significant differences in TA system expression between the exponential and stationary growth phases, with stationary-phase biofilms exhibiting higher levels of persister cell formation when exposed to ciprofloxacin and colistin. These findings highlight the clinical challenges posed by persisters, particularly in chronic infections dominated by biofilms and stationary-phase bacterial populations. Unlike VBNC bacteria, persister cells retain the immediate ability to regrow and cause recurrent infections following antibiotic treatment (Dawson et al., 2011). After the removal of antibiotic stress, persister cells typically regrow on nutrient media after a brief lag period, whereas VBNC cells do not grow immediately upon stressor removal, such as extreme temperatures (cold at 4°C or heat at 42°C), antibiotics, hypoxia, salinity, pH fluctuations, starvation, desiccation, or anaerobiosis (Ayrapetyan et al., 2015a; König et al., 2023). Some species of VBNC bacteria require specific treatments and longer periods for resuscitation after stress is removed. This is due to the time needed to repair essential proteins, restore TA ratios, and regain metabolic competence (Li et al., 2014). *In vitro* studies have used gradient centrifugation to distinguish between low- and high-density populations following heat shock treatment (Bruhn-Olszewska et al., 2018). The low-density population, typical of VBNC cells, requires much more time to resuscitate and regain its ability to grow on media. In contrast, the high-density population corresponds to persister cells, which can immediately resume growth upon transfer to the Luria broth (LB) medium, indicating their ability to recognize favorable conditions and resume growth. The depth of dormancy in VBNC cells and the time required for resuscitation are also influenced and regulated by the balance of free toxins within the cell (Andryukov et al., 2019). For instance, the high expression of antitoxins prevents cells from entering the VBNC state, while toxins such as RelE, ChpAK, and HipA in *E. coli* contribute to VBNC cell formation (Korch and Hill, 2006; Rotem et al., 2010).

The infectivity of the VBNC state remains an area of active research and debate (König et al., 2023). Some studies suggest it can be a transient phase, where bacteria later become cultivable and infective again, while others propose that it could represent a more persistent and dangerous reservoir for infection. Bacteria in the VBNC state can retain virulence, resistance to drugs, or multidrug treatments, making them potentially harmful to hosts (Ramamurthy et al., 2014). Therefore, VBNC cells differ from persisters in their measurable metabolic activity and prolonged resuscitation requirements, often requiring specific conditions to regain growth. The TA system plays a key role in regulating VBNC dormancy and recovery. VBNC cells pose notable challenges in public health due to their potential for antibiotic resistance and virulence and for causing chronic infections.

## 5 Resuscitation in the VBNC state

Under ideal conditions and after specific treatments, VBNC cells can revert to an active, cultivable state, a process known as “resuscitation.” (Bao et al., 2023). This term was first used to describe the recovery of non-cultivable *Salmonella enteritidis* cells following the addition of heart infusion broth. Baffone et al. further defined it as the reversal of the metabolic and physiological changes that characterize VBNC cells (Baffone et al., 2006). VBNC state cells pose hidden risks to public health, especially in food and waterborne diseases, making it crucial to understand their resuscitation process. Most VBNC-related studies have focused on the formation mechanism, while research on resuscitation mechanisms is limited. Resuscitation varies throughout bacterial species. For instance, the resuscitation of VBNC *S. typhimurium* cells varies based on the induction method, strain type, and environmental conditions. *S. typhimurium* could be resuscitated into cultivable cells only when administered orally, suggesting that the intestinal environment plays a key role in this process. However, Habimana et al. showed that the same strain failed to resuscitate after passing through the gastrointestinal tract (Habimana et al., 2014). Differences in strain types, animal characteristics, and the specific conditions that induce the VBNC state may contribute to these contrasting results. Understanding these variations requires further research to clarify the mechanisms influencing the resuscitation and pathogenic potential of VBNC cells. The resuscitation window in bacteria in the VBNC state differs significantly between species. While the time required to induce bacteria to enter the VBNC state varied and ranged from 5 min to 10 days (Mizunoe et al., 2000; Sun et al., 2008; Dinu and Bach, 2011; Ramamurthy et al., 2014; Robben et al., 2018; Wei and Zhao, 2018; Kan et al., 2019; Hamabata et al., 2021), Li et al. indicated that the duration of the resuscitation window may last from 3 days over months to 11 years (Li et al., 2014). Resuscitation can be driven by factors such as resuscitation-promoting factors (Rpfs), quorum sensing (QS), pyruvate sensing, ideal temperature, as well as various chemical agents such as Tween 20, Tween 80, NaCl, amino acids, vitamins, autoinducers, and antioxidizing compounds (Jia et al., 2020; İzgördü Ö et al., 2022; Pan and Ren, 2022).

The discovery and application of Rpf is a key advancement in VBNC cell resuscitation. Rpf is a muralytic enzyme found in high G+C gram-positive bacteria, promoting the growth of the VBNC state by breaking down bacterial peptidoglycan. The RpfB protein forms a complex that cleaves peptidoglycan during cell division—this process is essential for VBNC state resuscitation (Mukamolova et al., 2006). QS is a bacterial communication system that depends on cell density-dependent signaling, leading to coordinated changes in gene expression when populations reach a critical density (Abisado et al., 2018). QS involves the production of autoinducers (AI), such as AHL and AI-2, which trigger gene expression changes. This system aids bacterial adaptation to stressful conditions, including resuscitation of VBNC cells. AI-2 also activates antioxidant responses, such as catalase production, helping bacteria survive stress and regain cultivability (Ayrapetyan et al., 2014). Kong et al. linked the resuscitation of *Vibrio vulnificus* from the VBNC state to reduced catalase activity, which increases susceptibility to reactive oxygen species (Kong et al., 2004). Catalase production is restored during resuscitation, facilitated by the sigma factor RpoS, essential for catalase production (Smith and Oliver, 2006; Coutard et al., 2007; Ayrapetyan et al., 2014). QS inhibitors, such as cinnamaldehyde, can block QS signaling, controlling VBNC state resuscitation. Cinnamaldehyde disrupts QS pathways by inhibiting LuxR, delaying VBNC cell resuscitation (Brackman et al., 2008; Muehler et al., 2020). Sodium pyruvate (SP), an intermediate in glycolysis, plays a key role in the resuscitation of VBNC cells by aiding their growth under oxidative stress from H_2_O_2_ (Imazaki and Nakaho, 2009). In *E. coli*, SP helps cells return to a cultivable state after entering the VBNC state due to starvation at low temperatures. Similarly, *P. gingivalis* in the VBNC state under oxidative stress can be resuscitated by SP, which restores cultivability and viability (Progulske-Fox et al., 2022). SP serves as both a H_2_O_2_-degrading compound and a carbon source, supporting macromolecule biosynthesis and metabolic recovery in VBNC cells (Vilhena et al., 2019). In addition, cold stress can induce the VBNC state in *V. cholerae*, *Vibrio parahaemolyticus*, and *Vibrio vulnificus*, with resuscitation occurring once the stress is removed (Yoon and Lee, 2020). In *V. parahaemolyticus*, cells grown at 37°C transferred to starvation media at 4°C for 16 days entered the VBNC state, indicated by colony counts dropping below 1 CFU/ml by day 12. Non-cultivable cells resuscitated on agar media with catalase or sodium pyruvate formed colonies after 24 h at 37°C, although effectiveness decreased over time (Mizunoe et al., 2000). In addition to all of the aforementioned factors, most successful methods for resuscitating *C. jejuni* cells from a VBNC state have involved inoculating animals like chicks, chicken embryos, or suckling mice (Chaveerach et al., 2003; Baffone et al., 2006). However, to avoid animal use, researchers proposed *in vivo* chicken conditions using an immortal chicken cell line, LMH (Van et al., 2017), which is then used to resuscitate *Campylobacter hepaticus* VBNC cells (Phung et al., 2022). Resuscitating the VBNC state to an active cultivable state requires specific stimuli, such as temperature adjustments or chemical agents. Resuscitation varies widely among bacterial species in both required conditions and duration, with some taking minutes and others potentially spanning years. Understanding these dynamics is critical for addressing the persistence and reactivation of VBNC bacteria.

## 6 Significance of the VBNC state in public health

### 6.1 Medical context

While being frequently studied in the environmental and food industries, microorganisms in the VBNC state have also been observed in medical facilities on occasion due to VBNC state-associated microbial capacity to produce toxic compounds, thereby triggering threats to human health (Liu et al., 2017). The VBNC state has been observed across many bacterial species, explored in both medical and nonmedical contexts, such as food and water. Initial evidence of VBNC pathogenicity was shown using *V. cholerae* O1 in the rabbit ileal loop assay and was later confirmed in human studies, highlighting the potential public health risks (Amel et al., 2008). **Table 1** lists some of these bacterial species. A meta-analysis evaluated the frequency of non-cultivable bacteria in radicular periapical cysts, periapical granulomas, and periapical abscesses and found that VBNC cells most likely exert a major influence on the development and healing of these pathologies (Altaie et al., 2021). Behera et al. analyzed 97 culture-negative postoperative surgical site infection (SSI) samples using molecular tools, identifying bacterial pathogens in 53 using 16S rRNA gene PCR. The pathogens included *Bacillus* spp., *Pseudomonas* spp., *Enterococcus* spp., and other VBNC bacteria, which are slow-growing or hard to cultivate. This study highlights the challenges in detecting bacteria in SSIs that do not grow in conventional cultures due to factors such as antibiotic treatment or biofilm formation (Behera et al., 2021). Similarly, Wawrzyk et al. used high-throughput sequencing following vaporized hydrogen peroxide (VHP) decontamination at 300 ppm for 20 min to identify diverse microorganisms, including *Pseudomonas* spp., *Staphylococcus* spp., and *Aspergillus* spp., in a VBNC state on porous surfaces in an oral surgery clinic. The average concentrations were 8.0 × 10² CFU/m³ for bacteria and 6.3 × 10² CFU/m³ for fungi. Repeated VHP treatments had a minimal impact on the structural or chemical properties of the materials (Wawrzyk et al., 2020). VBNC pathogens can resuscitate in the human body, maintaining their virulence and posing a continued threat to food safety. Recent studies suggest that 80% of illnesses result from unidentified agents, possibly VBNC pathogens (Zhao et al., 2017). Research conducted on pathogenic *E. coli* and *Lactobacillus* spp. revealed that virulence genes were continuously expressed, and lactic acids were produced while the cells transitioned into the VBNC state (Liu et al., 2018; Lv et al., 2020). The 2011 *E. coli* O104:H4 outbreak in Germany highlights the VBNC state, where fenugreek sprouts and seeds were suspected sources of contamination, although the outbreak strain was rarely found in these foods. The strain likely entered the VBNC state due to environmental stress, such as exposure to copper ions, saline, and tap water from different regions. The bacteria remained viable for over 40 days under certain conditions, but cultivability decreased, with no colony-forming units detected after 3–5 days. Despite this, some bacteria retained intact membranes, indicating that they were still viable in the VBNC state. This study showed that VBNC *E. coli* O104:H4 could resuscitate in the human body, potentially leading to disease after food contamination (Aurass et al., 2011). These findings underscore that VBNC microorganisms, prevalent in clinical environments, pose marked threats to human health due to their ability to retain virulence and resist standard culturing methods. They contribute to infections and can be found on surfaces in medical settings, complicating infection control efforts.

**Table 1:** Research on VBNC state in microorganism.

Microbial species	Type of the diseases	Sources of the VBNC presence	Detection method or Techniques used	References
*P. gingivalis* and *A. actinomycetemcomitans*	Periodontitis	Oral cavity	Culture and qPCR	(Kozarov et al., 2005)
*E. faecalis*	Periodontitis	Oral cavity	Culture, PCR and histological	(Sedgley et al., 2005)
*E. faecalis*	Periodontitis	Oral cavity	Incubation at 4°C under direct light + room temperature without direct illumination Then, LIVE/DEAD kit, adherence assay, and biofilm formation method for detection of VBNC.	(Lleo et al., 2007)
*P. gingivalis*	Periodontitis	Oral cavity	Culture and microscopy	(Li et al., 2008)
*S. mitis, S. salivarius and S. sanguinis, S. mutans, P. gingivalis, F. nucleatum, Parvimonas micra, S. intermedius* and *A.actinomycetemcomitans*	Dental caries Periodontitis	Oral cavity	Safranin-staining, viable counts and microscopic	(Standar et al., 2010)
*S. enterica* and *C. jejuni*	Diarrhea, fever and abdominal cramps	Food	PMA-PCR	(Banihashemi et al., 2012)
*E. coli* and *B. subtilis* and viruses (MS2 and murine norovirus)	Gastrointestinal diseases	Food	PMA-qPCR	(Kim and Ko, 2012)
*S. epidermidis* and *S. aureus*	Opportunistic infections	Biofilms from central venous catheters	PMA-qPCR	(Zandri et al., 2012)
*S. oralis*, *S. gordonii*, *V. parvula*, *F. nucleatum* and *P. intermedia*	Dental caries Periodontitis	Oral cavity	PMA-qPCR	(Alvarez et al., 2013)
*E. faecalis*	Opportunistic infections	Water	PMA-qPCR	(Gin and Goh, 2013)
*S. cerevisiae*	Gastrointestinal diseases	Food	Flow cytometry and culture	(Salma et al., 2013)
*S. aureus* in biofilms	Opportunistic infections	Chronic implant-associated infections	Different concentrations of vancomycin or quinupristin/Dalfopristin + Nutrient depletion until loss of **c**ultivability	(Pasquaroli et al., 2013)
*S. Enteritidis*	Diarrhea, fever and abdominal cramps	Food	Confocal laser-scanning microscopy Flow cytometry	(Morishige et al., 2015)
*E. faecalis*	Periodontitis	Oral cavity	SYTO9+PMA-qPCR	(E et al., 2015)
*L. pneumophila* and *E. coli*	Diarrhea, fever and abdominal cramps	Water	LIVE/DEAD flow cytometry PMA-qPCR	(Zacharias et al., 2015)
*L. pneumophila*	Opportunistic infections	Water	Flow cytometric analysis	(Buse et al., 2013)
*E. coli*	Gastrointestinal diseases	Water	PMA-qPCR	(Kibbee and Örmeci, 2017)
*H. pylori*	Gastrointestinal diseases	Water	PMA-qPCR	(Orta de Velásquez et al., 2017)
*H. pylori*	Gastrointestinal diseases	Water	Sodium hypochlorite treated samples were exposed to PMA	(Moreno-Mesonero et al., 2017)
*H. pylori*	Gastrointestinal diseases	Water	LIVE/DEAD staining and Biology phenotype metabolism arrays (like Microarray plates PM1)	(Boehnke et al., 2017)
*V. cholerae*	Gastrointestinal diseases	Water	PMA-qPCR	(Wu et al., 2015)
*E.coli* O157:H7	Gastrointestinal diseases	Food	PMA-LAMP	(Yan et al., 2017)
*S. aureus*, *B. cereus*, *C. perfringens*, and Enterobacteriaceae	Gastrointestinal diseases	Food	PMA-qPCR	(El-Aziz et al., 2018)
*A. citrulli*	Gastrointestinal diseases	Food	Different concentrations of copper sulfate	(Kan et al., 2019)
*P. aeruginosa*	Opportunistic infections	Water	PMA-qPCR	(Golpayegani et al., 2019)
*R. biphenylivorans*	Gastrointestinal diseases	Food	First, VBNC state induced by norfloxacin, Then, VBNC state was detected by infrared spectroscopy	(Jia et al., 2020)
*L. monocytogenes*	listeriosis	Food	EMA and PMAxx-qPCR	(TruChado et al., 2020)
*C. jejuni*	Gastrointestinal diseases	Food	PMA-qPCR	(Lv et al., 2020)
*S. enterica*	Diarrhea, fever and abdominal cramps	Food	First, nutrition starvation, salt stress, low-level acidity, and low temperature. Then, PMA-PCR	(Wu et al., 2020)
*E.coli* O157:H7	Gastrointestinal diseases	Food	PMA-CPA	(Zhou et al., 2020)
*E.coli* O157:H7 and *S. enterica*	Gastrointestinal diseases	Food	PMA-qPCR PMA-LAMP	(Ye Z. et al., 2020)
*S. aureus*	Gastrointestinal diseases	Food	PMA-PCR	(Li et al., 2020b)
*S. enterica*	Diarrhea, fever and abdominal cramps	Food	PMA-CPA	(Ou et al., 2021)
MRSA	Gastrointestinal diseases	Food	PMA-CPA	(Jiang et al., 2021)
*P. acidilactici*	Gastrointestinal diseases	Food	PMA-CPA	(Guan et al., 2021)
*Cronobacter sakazakii*	Gastrointestinal diseases	Food	IMS + improved PMAxx ddPCR	(Lv et al., 2021)
*H. pylori*	Gastrointestinal diseases	Food	PMA and PEMAX™-qPCR	(Hortelano et al., 2021)
*P. gingivalis*	Periodontitis	Oral cavity	Exposure to hydrogen peroxide Then, LIVE/DEAD staining	(Progulske-Fox et al., 2022)
*E. coli*	Gastrointestinal diseases	Water	BacLight kit and fluorescence spectroscopy	(Wang et al., 2022)
*V. parahaemolyticus*	Gastrointestinal diseases	Food	PMA-qPCR	(Di Salvo et al., 2023)
*A. baumannii*	Opportunistic infections	Food	LIVE/DEAD staining, flow cytometry, respiratory activity assays, and resuscitation experiments	(König et al., 2023)
*C. jejuni*	Gastrointestinal diseases	Food	PMA-qPCR	(Reichelt et al., 2023)
MRSA	Gastrointestinal diseases	Food	PMA-qPCR	(Ou et al., 2021)
*S. aureus*, *S. epidermidis*, and *S. lugdunensis*	Opportunistic infections	Prosthetic joint infections	“Bacteriophages” First, gentamycin concentrations for VBNC state induction Then, VBNC state was detected by bacteriophage K and assessed in a qPCR	(Šuster and Cör, 2023)
*E. coli*	Gastrointestinal diseases	Food	Temperature, metal, and antibiotic	(İzgördü Ö et al., 2024)
*E. faecalis*	Periodontitis	Oral cavity	PMA-qPCR	(Sterzenbach et al., 2024)
*C. concisus*	Periodontitis	Oral cavity	Incubation at 4°C Then, PMAxx-qPCR	(Wahid et al., 2024)
*A. hydrophila*	Hemorrhagic septicemia in aquatic animals	Water	Plate count method and direct viable count microscopical method after staining with fluorescein diacetate and ethidium bromide	(Rahman et al., 2001)
*A. tumefaciens* and *R. leguminosarum*	Infectious disease in plants e.g. crown gall disease	Water	LIVE/DEAD staining	(Alexander et al., 1999)
*A. calcoaceticus, B. cepacia, and P. putida*	Waterborne diseases like diarrhea	Water	Colony hybridization and fluorescent *in situ* hybridization	(Lemke and Leff, 2006)
Thermophilic *Campylobacter* spp. (like *C. coli* and *C. lari*)	Gastrointestinal diseases	Water	Cultural and microscopic techniques	(Thomas et al., 2002)
*C. jejuni* and *E. coli*	Gastrointestinal diseases	Water	Cultural and microscopic techniques	(Cook and Bolster, 2007)
*C. freundii*	Gastrointestinal diseases	Water	Cultural and qPCR techniques	(Dhiaf et al., 2008)
*S. marcescens, K. planticola*, and *C. allerginae*	Gastrointestinal diseases	Water	Cultural and microscopic techniques	(Heidelberg et al., 1997)
*K. pneumoniae, E. aerogenes, A. tumefaciens, E. faecalis, M. flavus, B. subtilis*, and *Pseudomonas* strains	Gastrointestinal diseases	Water	Plate counts, acridine orange direct counts, and direct viable counts	(Byrd et al., 1991)
*E. hirae* and *E. faecium*	Opportunistic infections	Water	Cultural and qPCR techniques	(Lleò et al., 2001)
*H. pylori*	Freshwater health hazard	Water	LIVE/DEAD staining	(Adams et al., 2003)
*M. tuberculosis*	Tuberculosis	Soil and mammals	Starvation, oxygen limitation whole-genome sequencing	(Downing et al., 2005)

VBNC, Viable but non-cultivable; PCR, Polymerase chain reaction; PMA, propidium monovazide; CPA, priming amplification; IMS, Immunomagnetic separation; ddPCR, droplet digital PCR; qPCR, quantitative real-time PCR; *S. mitis, Streptococcus mitis; S. salivarius, Streptococcus salivarius; P. micra, Parvimonas micra; S. intermedius, Staphylococcus Intermedius; B. subtilis, Bacillus subtilis; S. oralis, streptococcus oralis; S. gordonii, streptococcus gordonii; V. parvula, Veillonella parvula; S. cerevisiae, Saccharomyces cerevisiae; B. cereus, Bacillus cereus; C. perfringens, Clostridium perfringens; A. citrulli, Acidovorax citrulli; P. acidilactici, Pediococcus acidilactici; C. sakazakii, Cronobacter sakazakii; S. lugdunensis, Staphylococcus lugdunensis*.

### 6.2 Food and environment context (nonmedical)

The presence of VBNC cells in food poses a risk to public health and food safety, as these cells are often undetectable by conventional methods. Factors such as low-temperature storage, pH, temperature, and pasteurization can induce bacteria into the VBNC state, where they remain dormant but retain pathogenic potential (Ferro et al., 2018; Schottroff et al., 2018). Strong evidence shows that VBNC cells in foods such as juice, milk, and beer can lead to contamination and make bacteria difficult to detect (Firmesse et al., 2012; Fakruddin et al., 2013; Muehler et al., 2020; Zhou et al., 2020). Xu et al. identified VBNC *Pediococcus damnosus* cells in spoiled beer using flow cytometry, routine culturing, and PMA-PCR to distinguish between cultivable and VBNC states. Genomic sequencing further confirmed that the isolates were exclusively *P. damnosus*. VBNC cells could be resuscitated using MRS agar with catalase, and their spoilage capabilities were similar to normal and resuscitated cells, posing a notable threat to food safety and preservation (Xu et al., 2021). Recent studies have detected *C. hepaticus* DNA in environmental sources on poultry farms, although it could not be cultivated from these samples (Phung et al., 2019). To explore *C. hepaticus* survival in farm environments, Phung et al. investigated its persistence in water and its transition to a VBNC state under stress (Phung et al., 2022). VBNC cells were induced by incubating *C. hepaticus* in Ringer’s solution at 4°C for 65 days. Resuscitation was attempted by coculturing VBNC cells with LMH, a chicken epithelial cell line, at 37°C for 48 h, followed by plating on HBA to detect recovery. Findings showed that *C. hepaticus* survived 3–4 days in water at 25°C and 21 days at 4°C. In the isotonic Ringer’s solution, survival increased to 9 days at 25°C and 64 days at 4°C. While optimal drinking water temperatures for poultry are around 23°C, lower water temperatures could allow longer survival, posing biosecurity risks and potential disease outbreaks. Nonthermal plasma (NTP) technology also offers a sustainable method for food decontamination, effectively inducing the VBNC state in *S. aureus* through metabolic suppression and oxidative stress responses. Liao et al. found that VBNC *S. aureus* exhibits enhanced resistance to oxidative stress, linked to the upregulation of antioxidative genes like *dps*, *trxA*, and *katA*. While heat, acid, and osmotic stress tolerance are similar between VBNC and cultivable cells, VBNC *S. aureus* shows greater infectivity and antibiotic resistance due to reduced cellular energy and overexpression of multidrug efflux pumps. Additionally, VBNC cells evade immune detection by downregulating pattern recognition receptors (PRRs) to persist longer (Liao et al., 2021). Furthermore, *E. coli* O157:H7 can enter a VBNC state in various water types, including river water and chlorinated drinking water. Liu et al. demonstrated that VBNC cells retain the ability to express both stx1 and stx2 genes, as confirmed by real-time PCR, enzyme-linked immunosorbent assays (ELISA), and Vero cytotoxicity assays. The expression of toxin genes in VBNC cells is higher than in cultivable cells, underscoring the potential pathogenicity of VBNC *E. coli* O157:H7 under stress conditions. This highlights the need for monitoring VBNC pathogenic bacteria, as they may still pose health risks despite not being cultivable (Liu et al., 2010).

Foodborne bacteria can form biofilms and enter a VBNC state, significantly affecting food safety and quality. The QS system, including molecules like AI-2 and Rpf, regulates biofilm formation, VBNC induction, and resuscitation, especially under stress conditions such as low temperatures, high osmotic pressure, and preservatives, making QS inhibition a promising strategy (Wu et al., 2020; Ding et al., 2024). In addition to the QS system, the VBNC state in foodborne pathogens is regulated by genetic mechanisms, including the stringent response mediated by (p) ppGpp, TA systems, and regulatory proteins like Lon/Clp proteases, RpoS, and OxyR (Wu et al., 2020). The stringent response mediated by (p) ppGpp helps bacteria adapt to stress by slowing growth and triggering VBNC state formation. The TA system regulates stress adaptation and biofilm formation, while key proteins like RpoS (Kusumoto et al., 2013) and Rpf (Ye Z. et al., 2020) play crucial roles in resuscitation, promoting growth and restoring pathogenicity. These factors, along with molecules like amino acids, contribute to the recovery of cultivability and virulence, complicating the management of VBNC bacteria in food processing (Zhu et al., 2024).

Organic acids, such as formic acid (FA), acetate, propionate, and butyrate, are widely used as food additives to block pathogens and improve gut health. Yadav et al. investigated the effects of FA on *Klebsiella pneumoniae*, *Acinetobacter baumannii*, two major hospital pathogens, at food storage temperatures between 4°C and 37°C (Yadav et al., 2022). FA treatment induced a VBNC state in these bacteria, with cells losing cultivability after 4 days but remaining viable for 10 days, as shown by flow cytometry. Interestingly, VBNC cells maintained membrane integrity, respiration, and smaller sizes, undergoing morphological changes from rods to shorter rods or cocci. The removal of FA resuscitated VBNC cells, increasing ATP levels and triggering the expression of virulence and antimicrobial resistance genes (ARGs). Resuscitation was successfully achieved using fresh and spent media.

Therefore, VBNC bacteria in food present notable risks to public health, as they retain pathogenicity and evade conventional detection methods. Stressful food processing conditions induce the VBNC state in pathogens like *E. coli*, *Salmonella*, and *Staphylococcus*, which can resuscitate in the human body and pose health threats. Studies reveal that genetic mechanisms, QS, and environmental factors regulate VBNC state formation and resuscitation. Understanding these factors is essential for controlling the risks posed by VBNC bacteria in food safety.

### 6.3 Biofilm and antibiotic resistance issues

In addition to sporulating bacteria, persister cells and VBNC bacteria exemplify phenotypic plasticity in microorganisms, enabling them to tolerate and eventually resist conventional antibiotic therapy (Andryukov et al., 2019). One of the most common forms of bacterial life occurs in biofilms, which, as mentioned, harbor antibiotic-resistant bacteria that are more challenging to eradicate than planktonic cell (Hu et al., 2019; Li et al., 2020a). The stressful microenvironments created by the complex structure of biofilms lead to physiological heterogeneity within the biofilm population. This heterogeneity likely supports the survival and maintenance of persister cells and VBNC bacteria (Orta de Velásquez et al., 2017). Despite being commonly associated with the biofilm phenotype, VBNC cells have also been reported in the planktonic populations of *S. aureus* and *E. coli* (Xu et al., 2018). Similarly, Gaio et al. evaluated VBNC state formation in planktonic cultures of *Staphylococcus epidermidis*. Their findings revealed that the proportion of VBNC cells can be modulated in both biofilm and planktonic conditions. Despite previous evidence that VBNC induction is strain dependent, recent findings suggest that VBNC cell formation can occur independently of the growth mode (Gaio et al., 2021).

*C. jejuni* can form monoculture biofilms or integrate into preexisting biofilms from strong biofilm producers like *Pseudomonas* spp., *Flavobacterium* spp., *Corynebacterium* spp., *Staphylococcus* spp., and *Enterococcus* spp (Ica et al., 2012). These biofilms are common in food processing environments, drinking water systems, and poultry houses. Cells within biofilms are more resistant to environmental stresses and disinfectants and survive under aerobic and low-temperature conditions longer than planktonic cells (Pitkänen, 2013). When detached from biofilms, *C. jejuni* can contaminate food products or water, posing considerable public health risks and contributing to its persistence in poultry facilities (Wingender and Flemming, 2011). *C. jejuni* can also enter the VBNC state under stressors like starvation, low temperature, and low pH. VBNC cells are more resistant to disinfection and can persist for up to 7 months. These cells may evade detection by culture-based methods but still express virulence genes, adhere to epithelial cells, and remain infectious. Both planktonic and biofilm-associated *C. jejuni* can transition to the VBNC state, with planktonic VBNC cells initiating biofilm formation on surfaces and contributing to contamination and persistence in various environments (Baffone et al., 2006; Trevors, 2011). Magajna et al. quantitatively assessed and compared the development of *C. jejuni* in planktonic and biofilm states using the LIVE/DEAD assay and traditional culturing methods (Magajna and Schraft, 2015). Biofilms were grown on glass fiber filters, while planktonic cells were cultivated in Mueller Hinton broth under microaerobic conditions at 37°C. Both were transitioned into the VBNC state by incubation at 4°C for up to 60 days, with viability assessed via LIVE/DEAD staining and periodic plate counts. Results showed that biofilm cells lost cultivability faster than planktonic cells, becoming VBNC within 10–20 days compared to 30–40 days for planktonic cells, likely due to gene expression differences. Mutants with altered polyphosphate kinase (Δppk1) formed more biofilm but were less capable of entering the VBNC state, linking biofilm formation, gene regulation (e.g., *csrA*), and the VBNC transition. Strain variations affect biofilm formation, VBNC state entry, and virulence. Clinical and food-processing isolates showed higher adhesion and virulence than animal isolates. This study highlights the need for nonculture-based detection methods to better understand the VBNC state and improve food safety to mitigate campylobacteriosis risks.

An *in vitro* study performed by Standar et al. demonstrated the role of VBNC cells in biofilm formation, highlighting their contribution to persistence and the chronic nature of infections. They investigate biofilm formation and interactions of *S. mutans*, *S. mitis*, and *Aggregatibacter actinomycetemcomitans* in mixed-species cultures (Standar et al., 2010). The results showed that cocultivating *S. mitis* with *S. mutans* increased biofilm mass, while cocultivating *S. mitis* with *A. actinomycetemcomitans* inhibited biofilm formation, suggesting that *S. mitis* can inhibit *A. actinomycetemcomitans* biofilm formation. In the latter group, the viable counting method failed to detect viable oral bacteria, suggesting that these bacteria could still contribute to biofilm mass without being considered “viable.” This observation implies that *A. actinomycetemcomitans* might adopt a VBNC status under specific conditions. Fluorescence microscopy combined with Live/Dead stain assays confirmed this, as both multiplying and VBNC cells were visualized as live cells. These findings highlight the role of VBNC cells in biofilm formation and persistence, underscoring their contribution to bacterial persistence and pathogenicity, even in the absence of typical growth.

While water system surveillance depends on culture-based techniques, many *Legionella* populations remain non-cultivable. A study revealed that starved VBNC *L. pneumophila* and *L. micdadei* can infect human macrophages and amoebae even after a year in ultrapure water. VBNC *Legionella* in oligotrophic biofilms may elevate bacterial concentrations in drinking water, resulting in underestimation of active cells using culture-based methods (Dietersdorfer et al., 2018). According to Ayrapetyan et al., VBNC *V. vulnificus* exhibits high-dose antibiotic tolerance, enabling it to withstand antibiotic treatment, heat exposure, heavy metals, pH fluctuations, and osmotic ionic challenges that would typically be lethal to bacteria (Ayrapetyan et al., 2018). Chlorine disinfection is widely used in drinking water treatment to ensure safety; however, the impact of residual chlorine on inducing bacteria in biofilms into a VBNC state remains unclear. Guo et al. investigated the cell numbers of *P. fluorescens* in different physiological states (cultivable, viable, and dead) using a heterotrophic plate count and flow cytometry under chlorine treatment. A significant difference between viable and cultivable cell numbers demonstrated that chlorine can induce bacteria in biofilms in the VBNC state. This study highlights the potential for bacteria to enter the VBNC state in drinking water biofilms and the changes in biofilm structure under chlorine treatment, providing important insights into biofilm control in drinking water distribution systems (Guo et al., 2023).

Biofilm-related infections can persist for long periods, contributing to chronic diseases and complicating the use of medical devices such as central venous catheters (CVCs) due to the reduced effectiveness of antibiotics on biofilm-growing bacteria. Concerns arise when bacterial clumps from mature biofilms spread through the bloodstream as septic emboli. Biofilm bacteria can enter a slow-metabolism form known as the VBNC state. A study by Zandri et al. found that 77% of CVC biofilms contained VBNC cells, primarily *S. epidermidis*. Viable cells were linked to febrile patients and positive blood cultures for *S. epidermidis*, suggesting that CVC biofilms act as reservoirs for staphylococci in the VBNC state (Zandri et al., 2012). Prosthetic joint infections (PJIs) pose significant treatment challenges due to the antibiotic resistance of infectious agents and their ability to form biofilms on surfaces. Weaver et al. explored the microbial species involved in PJI ecology in patients using both culturing and whole-genome shotgun sequencing (WGSS) techniques (Weaver et al., 2019). The results identified *P. aeruginosa* as the most abundant bacterium, and *B. fragilis* was detected exclusively by sequencing and could not be cultivated, likely due to biofilm resistance. In contrast, *S. aureus*, *E. faecalis*, and *Corynebacterium striatum* were successfully identified by culturing and sequencing. This study underscores the effectiveness of shotgun sequencing for detecting VBNC and culture-resistant bacteria, highlighting the limitations of traditional culturing methods, especially when microorganisms become resistant following biofilm formation. Biofilms are also significant for lactic acid bacteria (LAB), including food-related, probiotic, commensal, and pathogenic strains. During stress conditions like starvation or biofilm formation, some LABs enter a VBNC state. Under carbohydrate starvation, *Lactococcus lactis* was exposed to sterile chemically defined basal medium (CDM). For short-term starvation, CDM was supplemented with 0.1% lactose, while for long-term starvation, it contained 0.2% lactose or glucose. Under these conditions, *L. lactis* entered a VBNC state lasting at least two weeks, reducing DNA and protein synthesis while increasing glycolytic intermediate accumulation and the catabolism of alternative carbon sources. In addition, VBNC *L. lactis* shows enhanced expression of stress proteins, hydrolases, and peptidases, aiding survival without carbohydrates (Ganesan et al., 2007). The transition to a VBNC state was significantly influenced by time, carbohydrate type, and medium pH. Lower pH accelerated lactose metabolism and the onset of noncultivability. Strain-specific pH responses varied based on sugar utilization, indicating that carbohydrate use is the primary factor in starvation response and survival (Stuart et al., 1999).

**Figure 2:**
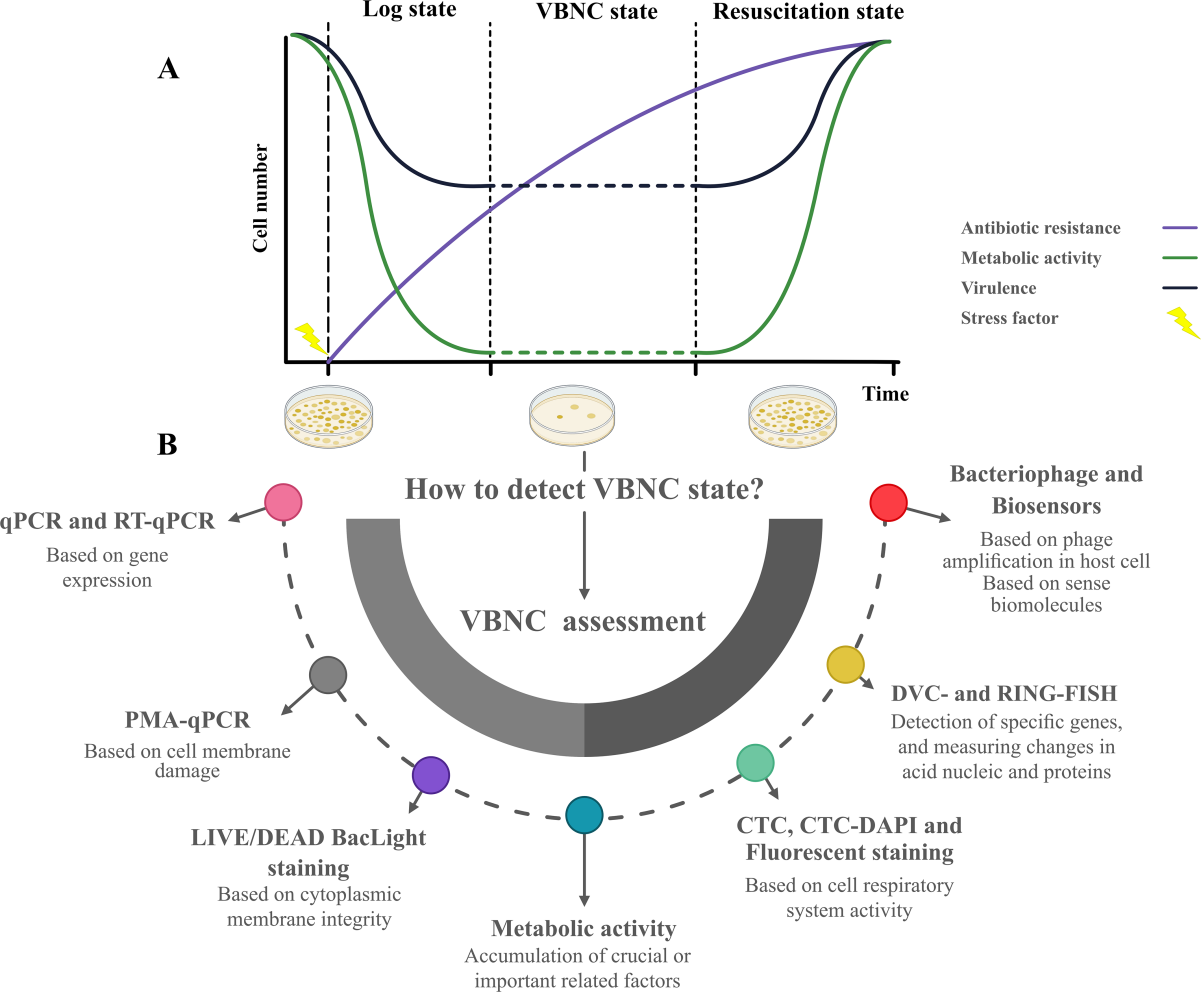
The life cycle of microorganism in Log state, VBNC state (in some studies also refer to Lag phase) and resuscitation state in terms of metabolic activity, virulence factor expression, and antibiotic-resistant situation **(A)**. Possible methods to assess the VBNC state when microbial species cannot be cultivated in medium and coexist with cultivable and dead populations **(B)**.

The microbial life cycle, illustrated in **Figure 2A**, focuses on three key states: the log phase, VBNC state, and resuscitation state, highlighting differences in metabolic activity, virulence factor expression, and antibiotic resistance. The VBNC state serves as a survival strategy under stress, where cells remain metabolically active but non-cultivable by conventional methods, complicating the detection of antibiotic failure (Rivers and Steck, 2001). Yadav et al. demonstrated the ability of *K. pneumoniae*, *A. baumannii*, and *E. coli* to transition into the VBNC state when exposed to ciprofloxacin, amoxicillin, or glutaraldehyde. This state was validated through confocal microscopy and assays assessing energy production, membrane integrity, and metabolism, highlighting the potential for VBNC cells in multidrug-resistant (MDR) nosocomial pathogens to contribute to surface contamination in hospital environments and emphasizing the need for less vulnerable antibacterial alternatives (Yadav et al., 2023). In a subsequent study, Yadav et al. further demonstrated that VBNC states induced by formic acid in *A. baumannii* and *K. pneumoniae* exhibit enhanced antimicrobial resistance and tolerance, driven by reduced metabolic activity, cellular changes, and the upregulation of outer membrane porin and antibiotic efflux pumps, mechanisms strongly associated with MDR. This adaptation enables these pathogens to withstand antimicrobial treatments in both hospital and nonhospital environments, such as food and pharmaceutical settings. The potential resuscitation of these pathogens poses a notable risk of environmental recontamination and the spread of resistant infections (Yadav et al., 2022).

Lin et al. demonstrated that chlorination in drinking water treatment can induce the VBNC state in *E. coli*, leading to increased bacterial persistence and antibiotic resistance. Low-dose chlorination reduced *E. coli* cultivability, leading to metabolic suppression and the upregulation of stress resistance genes (*rpoS, marA, ygfA, relE*) and antibiotic resistance genes (ARGs), particularly efflux-related ARGs. VBNC cells exhibited higher antibiotic efflux, resulting in lower intracellular concentrations. These findings highlight the persistence of VBNC *E. coli* in water systems, posing a risk of contamination and emphasizing the need for improved monitoring and control in water treatment practices (Lin et al., 2017). Postnikova et al. studied how *P. syringae* enters the VBNC state under oxidative stress from acetosyringone oxidation. After 3 h of exposure to H_2_O_2_, acetosyringone, and peroxidase, cultivability dropped by 99%, although membrane integrity was maintained. RNA sequencing showed upregulation of stress resistance genes (*rpoS* and *marA*), ARGs, and oxidative stress responses, while pathogenesis-related genes were downregulated. Transcription factors MarR and LysR indicated a shift from pathogenicity to survival. Efflux pump overexpression and drug inactivation mechanisms contribute to the VBNC state and antibiotic resistance, highlighting the persistence and resistance potential of VBNC cells (Postnikova et al., 2015). Bacteria such as *S. aureus* adapt to environmental stresses, such as antibiotics, by altering the FA composition (de Carvalho et al., 2009). Exposure to daptomycin increases membrane fluidity and surface charge (Jones et al., 2008), while vancomycin alters the phospholipid composition (Mirani and Jamil, 2013). Resistant strains convert saturated FAs to unsaturated FAs while lacking short-chain FAs. Gonçalves et al. exposed *S. aureus* cultures to vancomycin and teicoplanin, inducing dormancy and analyzing lipid composition. Most susceptible cells were eliminated, but a small fraction of the tolerant cells survived beyond 8 h. Fluorescence microscopy revealed more viable cells than CFU counts, and antibiotic-exposed cells decreased the branched/saturated FA ratio, reducing membrane fluidity (Gonçalves and de Carvalho, 2016). Consequently, antibiotic tolerance in the VBNC state might be a dynamic process that overlaps with persister cell tolerance mechanisms. Moreover, the presence of VBNC microorganisms in biofilm and clinical settings poses substantial health risks, since sterility is difficult to guarantee in everyday clinical practice.

## 7 Methods for detecting the VBNC state

As shown in **Figure 2B**, various methodologies for detecting and characterizing microorganisms were introduced for the VBNC state. Currently, viability assessments for detecting the VBNC state are categorized into membrane integrity, metabolism, and culture-based methods. These approaches are essential for studying microbial populations entering the VBNC state, but standard culture-based techniques fail to detect or differentiate cells. Understanding and assessing the VBNC state is crucial for addressing challenges in clinical microbiology, food safety, and environmental microbiology, as this state often contributes to persistent infections, contamination, and ecological resilience.

### 7.1 Culture-based methods

The public health significance of VBNC cells necessitates the development of reliable diagnostic techniques. However, conventional methods, such as culturing, are ineffective for accurately detecting VBNC cells in microbiological diagnosis. Robert Koch developed the conventional “plate culture method” in 1881 (Webb, 1932), and it has been extensively used for cultivating, identifying, and measuring alive microorganisms. Usually, at specific periods of time and temperature, viable bacteria produce colonies following the plating of a contaminated sample on an agar plate. Nonviable bacteria do not form colonies, which presents a major drawback of culture-dependent approaches in viability assays, as they fail to detect VBNC microorganisms (Trinh and Lee, 2022). Numerous studies have demonstrated that resuscitation of VBNC bacteria is generally more effective in broth cultures than on agar plates. This difference may arise from the distinct purposes of these media, with agar primarily serving for isolation while broth facilitates the preculture conditions needed for colony production. Effective resuscitation often requires specific conditions tailored to the microorganism and the factors underlying its noncultivability, highlighting the importance of optimizing environmental parameters for the successful recovery of VBNC cells (Özkanca et al., 2009).

### 7.2 Approaches based on metabolic activity

A viability assessment based on metabolic activity evaluates the biochemical processes within a cell or organism to determine its functionality and life status. This method measures indicators such as substrate consumption (e.g., glucose uptake) or byproduct production (e.g., oxygen usage or ATP generation), providing insight into metabolic health. Commonly used in cell culture and microbiology, this approach assesses cell viability and health without relying solely on traditional cell counting techniques, offering a dynamic perspective on cellular activity. The “direct viable counting (DVC)” method was developed to detect VBNC cells (Gonçalves and de Carvalho, 2016) and has been enhanced with radiolabeled substrates for microautoradiographic analysis to refine bacterial survival assessment in laboratory microcosms. By adding nutrients and nalidixic acid to inhibit cell division, the method distinguishes between cultivable and non-cultivable cells. Viable cells, including those undetectable on routine media, are identified as elongated cells, enabling accurate enumeration of responsive populations. Using this technique, VBNC cells of *V. vulnificus*, *C. jejuni*, *V. cholerae*, and *S. enteritidis* have been detected (Roszak and Colwell, 1987; Lv et al., 2020). Integration with advanced techniques such as direct fluorescent antibody incubation (DFADVC) (Mishra et al., 2011) or fluorescence *in situ* hybridization (FISH-DVC) (Piqueres et al., 2006) further enhances its precision in studying bacterial viability and behavior. The dye uptake assay is a metabolic activity-based viability method that quantifies dye absorption by viable bacteria through their membranes. In active bacterial systems, enzymes such as lipases, proteases, and esterases hydrolyze the dye as it enters the membrane, converting nonfluorescent signals into visible fluorescent signals (Trinh and Lee, 2022). Another metabolic activity-based viability method is the glucose uptake assay, wherein live bacteria take up and incorporate glucose from their environment into their cytoplasm via membrane transport systems, using it to produce energy through metabolic processes (Sundar et al., 2018). The glucose content within cells can serve as a valuable indicator for assessing bacterial metabolic activity. Two primary approaches for “glucose-based viability assessment” are enzymatic tests and artificial fluorescent glucose utilization. Notably, 2- [N-(7-nitrobenz-2-oxa-1,3-diazol-4-yl) amino]-2-deoxy-D-glucose (2-NBDG) is an artificial fluorescent glucose used in the artificial fluorescent glucose strategy to assess glucose uptake. Through a glucose transportation mechanism, only live bacteria with active metabolisms can metabolize 2-NBDG. The second strategy involves enzymatic tests to evaluate glucose uptake. Glucose is oxidized to produce H_2_O_2_ and D-gluconic acid in the presence of glucose oxidase. Next, o-dianisidine is used in a colorimetric process to quantify the H_2_O_2_ level. Peroxidase catalyzes this reaction by converting o-dianisidine from a colorless to a colorful molecule (Braissant et al., 2020).

Given that the VBNC state exhibits reduced metabolic activity compared to cultivable cells in the exponential phase (Li et al., 2014) and that VBNC bacteria cannot grow under standard culture conditions (Ayrapetyan and Oliver, 2016; Zhang et al., 2021), simultaneous detection of both viable and VBNC cells from the same sample is possible. Dead cells can be inferred based on decreased ATP synthesis rather than active recognition. Therefore, an “ATP production assay” is used to identify these cells (Fleischmann et al., 2021). This method combines a luciferase enzyme with bacterial lysis, which releases ATP and leads to fluorescein production. The level of fluorescence generated from this reaction reflects the ATP content within the cells, allowing for their detection (Robben et al., 2019). Robben et al. developed a VBNC–MIC assay using ATP production as a marker for bacterial viability to evaluate antimicrobial tolerance in VBNC bacteria. Heat-stress experiments showed that VBNC bacteria were resistant to antibiotics like ampicillin, ciprofloxacin, and gentamicin, as well as disinfectants like benzalkonium chloride. The assay validated VBNC induction through ATP production and cultivability tests and determined the minimum ATP inhibitory concentration (MAIC) for antimicrobials. Temperature-dependent time-kill experiments and fluorescence microscopy confirmed a strong correlation between ATP levels and bacterial viability, even under severe stress (Robben et al., 2019). This method provides a high-throughput, cost-effective approach to studying the antimicrobial resistance of VBNC bacteria and could advance the development of targeted treatments or disinfection strategies.

Metabolic-based approaches thus offer innovative tools for assessing the viability of VBNC bacteria by measuring metabolic activity markers, such as glucose uptake, ATP production, and dye hydrolysis. These methods enable differentiation between viable and nonviable cells, overcoming the limitations of traditional culturing techniques. Techniques like ATP production assays may also reveal antimicrobial resistance in VBNC bacteria, highlighting the need for advanced detection and treatment strategies to address their potential public health risks.

### 7.3 Approaches based on membrane integrity

The dye exclusion assay and molecular methods are widely used to evaluate bacterial viability based on membrane integrity. Bacteria with an intact membrane selectively exclude dyes, while those with compromised membranes are more permeable. The dye interacts with internal proteins and nucleic acids, leading to the release of measurable fluorescent signals (Breeuwer and Abee, 2000; Trinh and Lee, 2022).

#### 7.3.1 Dye exclusion and flow cytometry

Propidium iodide (PI) is widely used in dye exclusion experiments to assess bacterial membrane damage. PI penetrates bacteria with compromised membranes, binding to their RNA and DNA (Cieplik et al., 2018). This interaction increases PI fluorescence approximately 30-fold, shifting its excitation/emission maxima from 493/636 nm to 535/617 nm. The resulting fluorescence can be analyzed using flow cytometry (FCM), confocal laser scanning, or fluorescence microscopy, making it a key tool in microbial analysis (Stiefel et al., 2015). Combining PI with SYTO 9 (Cieplik et al., 2019) or SYBR Green (Muehler et al., 2020) in the LIVE/DEAD method offers an effective approach to assessing cell viability based on cytoplasmic membrane integrity. SYTO 9, a green fluorescent dye, stains all cells, whether intact or damaged, while PI, a red fluorescent dye, selectively penetrates cells with compromised membranes, competing with SYTO 9 for nucleic acid binding. This dual-staining technique allows for the differentiation of viable cells with intact membranes from nonviable, membrane-compromised cells (Boehnke et al., 2017). This method, often coupled with FCM or fluorescence microscopy, is particularly effective in detecting VBNC state cells, which maintain intact membranes (Zhao et al., 2013). Compared to DVC or CTC staining, the combination of FCM with PI and SYTO 9 enhances detection sensitivity and has become a widely regarded standard procedure (Santander et al., 2018). FCM is a powerful tool for rapid microbial enumeration, analyzing thousands of cells per second. When combined with fluorescent viability kits, it provides both quantitative and qualitative data and allows precise cell sorting. Fluorogenic substrates enhance detection by producing polar fluorescent products in cells with intact membranes, although their use can be limited by background interference from nontarget bacteria or particles when using fluorescently labeled antibodies or oligonucleotides (Khan et al., 2010). FCM has limitations, including its inability to distinguish VBNC cells from viable-cultivable ones or to differentiate between bacterial species, restricting its use for VBNC-related infections. Viability quantitative PCR (v-qPCR) methods like PMA-qPCR may be more effective for specific food and water matrices (Trinh and Lee, 2022).

#### 7.3.2 PCR-based techniques

Thanks to advancements in molecular biology, current molecular methods, such as polymerase chain reaction (PCR), are now better alternatives for species identification. However, the applicability of PCR for detecting the VBNC state is limited due to its inability to distinguish DNA from bacterial suspensions or agar media where cultivable, dead, and VBNC cells coexist. Bacteria with damaged membranes can be penetrated by DNA-intercalating dyes like ethidium monoazide bromide (EMA) and propidium monoazide bromide (PMA), but live bacteria with intact membranes are less susceptible to being visualized by dye-based methods (Kontchou and Nocker, 2019; Xie et al., 2021). For bacterial viability tests, PCR and loop-mediated isothermal amplification (LAMP) have frequently been used in conjunction with photoreactive DNA-intercalating dyes. Prior to extracting DNA from bacteria, cells are typically treated with PMA. PMA can attach to DNA and stop its replication after passing through the broken membrane. Quantitative PCR (qPCR) can then be used to quantify VBNC cells (Kibbee and Örmeci, 2017). To further stimulate the interaction between these dyes and DNA, the sample is subjected to visible light at 600 nm (Hein et al., 2007). The azide group in EMA and PMA changes into highly active nitrene radicals that bind to the DNA of the nonviable bacteria. Then, the DNA structure is altered and DNA elongation in PCR is inhibited. Due to the covalent bond formation between DNA and nitrene radicals, DNA from nonviable bacteria cannot be amplified, unlike DNA from viable bacteria (Taylor et al., 2014; Zhang et al., 2015; Lee et al., 2022). PMA-qPCR and PMA-LAMP have been widely explored for the viability assay for various bacteria entering the VBNC state (Golpayegani et al., 2019; Zhang et al., 2020; Xu et al., 2021). Moreover, a recently enhanced PMA dye, PMAxx, has been used for bacterial viability tests to increase selectivity and sensitivity (Cao et al., 2019; Gao et al., 2021). Reverse transcription-quantitative PCR (RT-qPCR) and droplet digital PCR (ddPCR) are other molecular diagnostic techniques used to quantify VBNC cells. RT-PCR requires a target gene that expresses consistently, such as virulence and housekeeping genes. Moreover, ddPCR is a relatively new technology considered more effective than other methods due to its independent amplification efficiency and lack of reliance on a calibration curve (Lv et al., 2020). Therefore, dye exclusion assays and molecular methods effectively assess VBNC state bacterial viability by evaluating membrane integrity. Flow cytometry with fluorescent dyes, such as PI and SYTO 9, differentiates viable and nonviable cells, while PCR-based methods like PMA-qPCR enhance specificity by targeting DNA in cells with damaged membranes. Despite certain limitations, these advanced tools offer precise and versatile solutions for microbial analysis across diverse fields.

### 7.4 Other techniques

Matrix-assisted laser desorption/ionization time-of-flight mass spectrometry (MALDI-TOF MS) offers a valuable technique for identifying VBNC bacteria by analyzing the unique protein profiles of cells, even when they are non-cultivable. This approach, often combined with multivariate data analysis, allows researchers to differentiate between viable and nonviable cells (Kuehl et al., 2011). For example, Heim et al. analyzed the protein expression patterns of *E. faecalis* in different exponentially growing, starved, and VBNC states to investigate whether the VBNC state was distinguishable from other stress responses. The results revealed that VBNC cells have a distinct protein profile compared to starved or growing bacteria, confirming that the VBNC state is a separate physiological phase activated in response to environmental stress. In the analysis, proteins were excised from Coomassie blue-stained gels, digested with trypsin, and analyzed using MALDI-TOF, with peptide extracts eluted and directly analyzed on the MALDI target. The data obtained were used for protein identification through searches in protein databases, providing insight into the unique characteristics of VBNC bacteria. The findings showed that the protein profile of VBNC cells differs significantly from that of starved or exponentially growing cells. This suggests that the VBNC state represents a distinct physiological phase in the life cycle of *E. faecalis*, one that is triggered in response to various environmental stresses (Heim et al., 2002).

RNA-based methods, such as 16S rRNA sequencing, targeting metabolically active VBNC state cells offer a solution. Guo et al. employed culture-dependent methods in combination with quantitative PCR using PMA dye to assess cellular viability. They also developed an innovative approach to quantifying viable pathogens by correlating specific gene copy numbers with viable cell counts. This approach revealed that the ratio of cultivable bacteria to viable 16S *rRNA* gene copies varied between water and biological activated carbon (BAC) biofilms (Guo et al., 2021). However, the rapid degradation of RNA poses challenges (Guo et al., 2021). The BrdU labeling technique developed by Malayil et al., which marks replicating DNA in metabolically active cells, was coupled with next-generation sequencing (NGS) to assess VBNC *Vibrio* spp. in water sources (Malayil et al., 2021). This study demonstrates that combining BrdU labeling with 16S rRNA sequencing effectively detects metabolically active VBNC *Vibrio* spp. in water samples. This method eliminates the need for enrichment steps, significantly reducing detection time. Taxonomic analysis identified Proteobacteria as the predominant phylum across samples, while beta diversity analysis indicated variations between BrdU-treated and nontreated samples. This study underscores the effectiveness of BrdU labeling in detecting VBNC bacteria and highlights its potential in monitoring water quality.

NGS techniques like metagenomics provide a highly sensitive and specific method for detecting and identifying difficult-to-culture microbes, including VBNC bacteria. Unlike targeted amplicon sequencing, shotgun metagenomics enables the functional annotation of gene sequences found in clinical samples, providing a broader and more detailed description of microbial communities. Functional annotation involves two key steps: first, gene prediction, where bioinformatics algorithms identify potential protein-coding sequences, and second, gene annotation, where these sequences are matched to protein family databases to determine their functions. This approach not only allows for the discovery of novel genes and functional pathways but also aids in identifying difficult-to-culture microorganisms like VBNC (Mellmann et al., 2011; Boers et al., 2019). This approach is particularly valuable for studying microbial communities where VBNC bacteria may be present in significant numbers, providing critical insights into their presence and potential viability (Boers et al., 2019).

The “DNase I protection assay” is another fascinating technique that relies on the protection of cellular genomic DNA from exogenous nuclease degradation. Unlike damaged cells with exposed nucleic acids, VBNC cells with intact membranes can survive and be identified using this method (Pawlowski et al., 2011). Phage-based methods aim to track labeled phages that bind specifically to bacterial hosts, amplify measurable markers within the host using the phage, and facilitate the proliferation of phage products released from the host. These techniques are valuable for identifying and monitoring bacterial populations, including VBNC cells (Ben Said et al., 2010; Smartt and Ripp, 2011). Biological sensors, which transform biological chemical signals into measurable electrical or visual outputs, provide an innovative method for detecting VBNC cells. Potentiometric sensors and functional polymer-based sensors selectively identify living aerobic and facultative anaerobic bacteria. Advanced techniques, such as piezoelectric immunosensors and mass-sensitive cantilever sensors, enhance detection speed and specificity for viable cells, although they remain complex, with detection times ranging from hours to a day (Koçak et al., 2023). Cheng et al. detected *E. coli* in the VBNC state using electrochemical sensors and electrodes harboring *Pseudomonas putida* and *Moraxella* spp. based on membrane specificity and *β*-D-glucuronidase activity (Cheng et al., 2011). The effectiveness of this procedure is determined by the minimum number of microorganisms detected within a given timeframe. For instance, Togo et al. demonstrated that *E. coli* was found in water samples within 20 min at as low a density as 2 CFU/100 mL using biosensors carrying *P. putida* and *Moraxella* spp (Togo et al., 2007). An “aptamer-based biosensor” found *S. typhimurium* in fewer than 600 colonies (Labib et al., 2012). Other approaches developed to detect VBNC bacteria include microfluidic-based techniques (Bamford et al., 2017; Vilhena et al., 2019), autoradiography (Lambrecht and Ulbrich-Hofmann, 1993), and D2O-labeled Raman spectroscopy (Guo et al., 2019).

In summary, viability assessments are critical markers utilized to precisely identify the presence of VBNC cells during induction and resuscitation. We identified several advantages and limitations of LIVE/DEAD staining and molecular assays for VBNC state detection. Both methods rely on membrane integrity; however, compared to PCR, LIVE/DEAD BacLight staining provides more precise differentiation between live and dead cells, especially when combined with FCM. In addition, EMA- and PMA-PCR, even with the drawback of false positives, are expensive and require a qualified technician (Fleischmann et al., 2021; Wideman et al., 2021). For effective and reliable detection of VBNC cells, a combination of different techniques seems worthwhile for achieving the best results. For instance, Xu et al. introduced a novel procedure to identify and confirm VBNC *P. damnosus* in spoiled beer, utilizing techniques like flow cytometry, routine culturing, and PMA-PCR. Genomic sequencing confirmed that these cells were identical to *P. damnosus*, with no contamination from other species. Then, VBNC cells were successfully resuscitated using MRS agar supplemented with catalase, and both the VBNC state and resuscitated cells retained their contamination capability. This approach provides a valuable framework for studying VBNC states in food safety, helping to identify and mitigate risks posed by VBNC microorganisms in the food industry (Xu et al., 2022). Moreover, RNA-based techniques such as 16S rRNA sequencing, NGS, and metagenomics offer rapid, sensitive, and specific tools for studying VBNC states and monitoring microbial communities in diverse samples.

## 8 VBNC state in the oral cavity

The oral microbiome is influenced by environmental factors such as pH, temperature, humidity, anaerobic conditions, nutrition, and hormone levels (Saadaoui et al., 2021). To persist in the oral cavity, most oral bacteria rely on biofilm formation for survival (Lemos et al., 2005). Oral biofilms are unique compared to those in other parts of the human body due to their specific location, dynamic nature, formation process, and composition, primarily involving plaque formation in dental hard tissues (Ramachandra et al., 2023). The oral cavity hosts diverse biofilms across various niches, comprising over 700 bacterial species, fungi, algae, protozoa, and viruses (Wade, 2021). These biofilms can either support oral health or contribute to disease. Commensal bacteria promote oral health by protecting tissues, preventing pathogenic attachment, and modulating immune responses (Gutt et al., 2018). Conversely, biofilms can facilitate the bacterial evasion of immune defenses and antimicrobial treatments, markedly contributing to antimicrobial resistance (Singh et al., 2017).

The oral cavity hosts numerous non-cultivable or culture-difficult bacterial species (Ye C. et al., 2020). Studies by Miller et al. and Socransky et al. revealed the limitations of traditional culture methods, showing that about half of the oral microbiome remains non-cultivable, for which studies have highlighted their role in periodontitis (Siqueira and Rôças, 2013). Metagenomics studies identified *Bacteroidetes* spp., *Prevotella* spp., *Treponema* spp., *Peptostreptococcus* spp., *Fusobacterium* spp., Eubacterium spp., *Filifactor alocis*, and *Parvimonas micra* as persisting species in the subgingival biofilm, especially after treatment like mechanical periodontal therapy combined with amoxicillin and metronidazole (Siqueira and Rôças, 2009; Colombo et al., 2012; Pérez-Chaparro et al., 2014). By generating gradients of nutrients, oxygen, and pH, biofilm-producing bacteria create localized stress conditions, such as hypoxia, that induce the VBNC state (Alam et al., 2007). QS and signaling molecules intensify bacterial stress responses and metabolic shifts, driving VBNC induction. Environmental stresses, including oxygen exposure, starvation, and osmolarity changes, further stimulate formation and morphological transitions linked to the VBNC state (Santos et al., 2023). Sub-lethal antimicrobial levels and oxidative stress activate survival pathways, while reduced metabolic activity and slower growth rates in biofilm-associated bacteria reflect VBNC characteristics (Bronowski et al., 2014).

Despite limited research on the VBNC state in oral bacteria, those within biofilms are expected to enter this state naturally in response to disinfectants or antibiotics. Progulske et al. showed that *S. mutans*, *S. pyogenes*, and *Streptococcus sanguinis* exhibit phenotypes similar to the VBNC state (Progulske-Fox et al., 2022). Bacteria can survive unfavorable conditions by entering the VBNC state, enhancing their resilience in the oral cavity. Biofilms act as reservoirs for these bacteria, supporting their persistence, contribution to recurrent infections and antibiotic resistance (Yahara et al., 2017; Progulske-Fox et al., 2022). The VBNC state also enhances biofilm resilience in endodontic infections by enabling bacteria to endure extreme stress and persist within the root canal system. *E. faecalis* is a biofilm-forming bacterium strongly associated with endodontic infections and root canal therapy failure. Its adaptability allows it to thrive in extreme conditions, including alkaline pH, salt-rich environments, and high temperatures. Structural components such as glycerol teichoic acid and peptidoglycan strengthen its cell membrane, resist osmotic pressure, and enhance its overall resilience (Jayakumar et al., 2024). This VBNC state is characterized by cell wall modifications that protect the bacterium and allow it to persist. Its small size further facilitates its invasion of dentinal tubules, where it uses virulence factors, such as collagen-binding proteins, to adhere to dentin and establish infection. Additionally, *E. faecalis* possesses a proton pump in its cell wall that helps regulate intracellular pH by acidifying its cytoplasm (Kayaoglu and Ørstavik, 2004). This mechanism is particularly advantageous in alkaline environments, such as those created by calcium hydroxide-based intracanal medicaments, ensuring their survival and persistence. These combined features make VBNC *E. faecalis* highly resilient and challenging in endodontic infections (Ran et al., 2015).

The limited research on oral bacteria’s ability to enter the VBNC state makes it difficult to understand its full implications for oral health. For example, the VBNC state in *P. gingivalis* may contribute to the persistence of periodontal diseases. Additionally, *H. pylori* in the oral cavity could be significant in gastroenterology, as it is linked to conditions such as ulcers and stomach cancers. VBNC bacteria can evade detection during routine culturing, resist antibiotics, and persist undiagnosed in the oral cavity. These bacteria may reactivate under certain conditions, leading to recurrent infections (Marginean et al., 2022; Progulske-Fox et al., 2022).

### 8.1 VBNC state in *P. gingivalis*, *F. nucleatum*, and *A. actinomycetemcomitans*

For many years, researchers were unable to cultivate periodontitis-associated bacteria from the oral cavity and atherosclerotic vessels, so they believed that detecting genomic DNA from oral pathogens in diseased tissues did not confirm the existence of the VBNC state but merely indicated the presence of DNA potentially transported by macrophages to the affected area (Kozarov, 2012). Studying this theory led to two key findings. First, Kozarov and colleagues examined whether viable *P. gingivalis* and *A. actinomycetemcomitans* were present in diseased tissues (Kozarov et al., 2005). Both bacteria were detected using qPCR, while attempts to cultivate live colonies on blood agar plates were unsuccessful. When carotid atherosclerotic plaque homogenate was introduced into human cardiovascular aorta endothelial cells, the authors were able to distinguish live *P. gingivalis* and *A. actinomycetemcomitans* from dead ones. Their presence in the plaque was confirmed using cell culture invasion assays and immunofluorescent microscopy. Since detection on blood agar does not confirm bacterial viability, this suggests that the bacteria remain alive within the host cells. Their viability, rather than their ability to be cultivated, may indicate their VBNC state. In a multispecies environment, such as a dual-species culture, interactions between *P. gingivalis* and *A. actinomycetemcomitans* may enhance the growth of both bacteria (Periasamy and Kolenbrander, 2009). However, as Kozarov et al. suggested, these bacteria might exist in a VBNC state within atherosclerotic plaque. Given their low metabolic activity in this state, specific growth conditions must be considered to facilitate their revival. These conditions may include the use of anaerobic environments or media enriched with nutrients and metabolic products, such as pyruvate, as Progulske et al. demonstrated for *P. gingivalis* resuscitation. Additionally, signaling molecules either naturally present or released by other metabolically active species could “wake up” or stimulate the bacteria to resume metabolism. These optimized conditions may improve the revival of these intracellular bacteria from the VBNC state, even when cultivated on blood agar.

This report raises the question of what happens to these organisms during *in vitro* and *in vivo* investigations. Haditsch et al. conducted an *in vitro* study demonstrating *P. gingivalis*’s ability to invade and persist in mature neurons, using the *in vitro* model to investigate its neurodegenerative effects. Their findings revealed that *P. gingivalis* infection remained stable for up to 72 h, during which time the bacterium transitioned into a VBNC state. Confocal high-content screening (HCS) and qPCR analysis confirmed sustained intra-neuronal infection, while CFU assays demonstrated a marked decline in cultivable bacteria over time. To validate the VBNC state, RNA analysis detected active transcription of key *P. gingivalis* genes, including lysine gingipain (*kgp*), arginine gingipain (*rgpB*), and 16S rRNA, at all time points. Intracellular *P. gingivalis* was localized within lysosome-like structures and unbound in the cytoplasm, with colocalization observed for endosomal and lysosomal markers. In addition, bacterial aggregates partially engulfed by neuronal membranes were shown to produce gingipains, suggesting that these clusters act as biofilm-covered reservoirs. These findings indicate that *P. gingivalis* can persist within neurons in a VBNC state, potentially serving as a chronic source of infection and contributing to neurodegenerative processes (Haditsch et al., 2020). Another study by Li et al. provides further evidence that *P. gingivalis* can enter the VBNC state as part of their life cycle. Their follow-up findings demonstrated that *P. gingivalis* can be detected for up to 48 h during cocultivation with a cell line. However, after 48 h, the number of colonies on blood agar plates drastically decreased, while intact *P. gingivalis* inside the cells could be visualized using a microscope. A number of colonies formed on blood agar plates after lysing infected cells and mixing them with uninfected cells (Li et al., 2008).

*F. nucleatum* is part of the oral anaerobic normal flora, often initiating as an oropharyngeal infection but also acting as a pathogen in abscesses across various organs (Kuppalli et al., 2012). The VBNC state, where bacteria are metabolically active but fail to grow under standard laboratory conditions, may explain the difficulty in recovering *F. nucleatum* using conventional culture methods. For example, Chakvetadze et al. reported a case where *F. nucleatum* could not be recovered from blood cultures or from hepatic, pleural, and brain drain fluid in a patient with multiple abscesses. The diagnosis was ultimately made through PCR targeting the 16S *rRNA* gene in brain abscess drain fluid, underscoring the importance of molecular techniques for diagnosing VBNC bacteria when standard cultures fail (Chakvetadze et al., 2017). *F. nucleatum* also maintained stable biomass and showed slight increases in superoxide dismutase activity when cultivated in a gradually oxygenated atmosphere, suggesting that this metabolic adaptation helps *F. nucleatum* survive in oxygenated environments, a feature linked to its ability to enter the VBNC state (Diaz et al., 2000).

Oral bacteria, including *Prevotella intermedia*, *P. gingivalis*, *F. nucleatum*, and *H. pylori*, can migrate to other organs via systemic circulation or swallowed saliva, with bacteremia increasing inflammation and potentially causing organ dysfunction (Chopra et al., 2024). While gastric acidity inhibits most oral bacteria, species such as *P. gingivalis* can survive harsh conditions, cross the gut barrier, and enter systemic circulation. This survival is partly attributed to the VBNC state observed in bacteria such as *P. gingivalis*, *H. pylori*, *A. actinomycetemcomitans*, and *E. faecalis*. The VBNC state enables these bacteria to evade detection or persist intracellularly, contributing to the chronicity of oral and systemic infections (Chopra et al., 2024). Understanding the molecular mechanisms behind the VBNC transition, particularly in *P. gingivalis*, is essential for managing periodontal diseases and their systemic impacts. Periodontitis-associated bacteria can remain viable in a VBNC state within host cells, evading conventional detection methods. Investigating their behavior in both *in vitro* and *in vivo* contexts is vital for uncovering their role in disease progression and developing effective management strategies.

### 8.2 VBNC state in oral *Enterococcus* spp., *Streptococcus* spp. and other oral bacterial strains

*Streptococcus mutans*, a key oral pathogen linked to dental caries, is a well-documented example of streptococcal dormancy. While the dormancy state in most streptococcal species is commonly triggered by antibiotics (Willenborg et al., 2014), the dormancy state in *S. mutans* is regulated by TA systems, and induced by environmental stressors such as dimethylaminododecyl methacrylate, oxidative stress, amino acid depletion, antibiotic challenge, acid stress, and heat (Leung and Lévesque, 2012). *S. mutans* causes dental caries by metabolizing sucrose to produce lactic acid, which erodes tooth enamel. *S. mutans* relies on the CSP-ComDE system to regulate both dormancy and biofilm formation where dormant cells are found (Senadheera and Cvitkovitch, 2008). Understanding the interplay between the CSP-ComDE system, dormancy, and biofilm formation is critical for developing effective treatments (Buse et al., 2013). In addition, *S. sanguinis*, a commensal organism in the human oral microbiota, utilizes a dormant state as a stress response. It resides in the oral cavity, where it suppresses the growth of caries-causing *S. mutans* by producing H_2_O_2_ as a metabolic byproduct (Aynapudi et al., 2017). Under specific conditions, *S. sanguinis* also promotes the formation of health-associated biofilms, such as dental plaques. Decker et al. documented that stationary-phase cultures of *S. sanguinis* develop a subpopulation of viable bacteria undetectable by plate count, suggesting the presence of a VBNC state during nutrient depletion (Decker, 2001). Group A Streptococcus (GAS) is a key pathogen in oral source septicemia and periodontal disease progression (Trapp and Scott, 2017). Under stress, *Streptococcus pyogenes* can enter a VBNC state, maintaining metabolic activity while evading standard detection methods (Wood et al., 2005). Trainor et al. were the first to report VBNC formation in *S. pyogenes* under nutrient-limited conditions, where carbon or phosphorus deprivation triggered a starvation state lasting 3–4 weeks, causing a decline in cell viability and noncultivability after 4 days. Amino acid utilization plays a key role in survival, and VBNC cells exhibit functional membrane potential but no growth on standard media. Active cell wall and protein synthesis are essential for maintaining viability in this dynamic state (Trainor et al., 1999). Wood et al. further explored *S. pyogenes*’ long-term survival, finding that, in sugar-limited Todd-Hewitt broth, the bacteria remained cultivable for over a year, while survival in glucose-rich or defined media lasted less than a week. After 4 weeks in sugar-limited conditions, VBNC cells with intact membranes were detected. These findings highlight the ability of *S. pyogenes* to persist for extended periods under specific nutrient-limited conditions, underscoring its adaptive strategies for long-term survival (Wood et al., 2005).

*S. mutans*, *S. sanguinis*, *Veillonella* spp., *Actinomyces* spp., *Bifidobacterium* spp., and *Lactobacillus fermentum* are linked to oral health, and some of these species are also associated with dental caries development. Kim et al. investigated *Limosilactobacillus fermentum* and *L. plantarum* persister cells under antibiotic stress (Kim et al., 2024). They found that *L. fermentum* cells treated with 4 μg/ml amoxicillin for 30 h formed persister cells, while *L. plantarum* treated with 400 μg/ml ampicillin for up to 60 h showed similar dormancy. The persister cells exhibited low resuscitation (0.5%–1%) and characteristics typical of dormancy, such as multi-antibiotic tolerance. Stress-induced ribosome inactivation led to dormancy in Lactobacillus. Unlike *E. coli*, *Lactobacillus* spp. lacks flagella, and its resuscitation rate is lower, possibly due to the simpler structure of its peptidoglycan-rich cell wall. Additionally, prolonged antibiotic treatment induced the VBNC state in *L. plantarum*, causing cells to lose cytoplasmic content and cell walls, rendering them unable to resuscitate. This suggests that persister formation and resuscitation mechanisms depend on bacterial characteristics, such as membrane structure. Bao et al. investigated the molecular composition of *Lacticaseibacillus paracasei* in a VBNC state using single-cell Raman spectroscopy, fluorescent microscopy, plate counting, and scanning electron microscopy (Bao et al., 2023). After cold incubation at 4°C for 220 days, the viable plate count was zero, but the live cells were still visible under fluorescence microscopy, indicating that the bacteria entered the VBNC state. SEM showed altered morphology with shorter cells and wrinkled surfaces. Raman spectra revealed significant biochemical differences, including changes in carbohydrates, lipids, nucleic acids, and proteins. These findings suggest that the VBNC state involves cellular structural and biochemical adaptations to adverse conditions, providing insights into VBNC state formation in lactic acid bacteria.

*E. faecalis* is also considered one of the most frequently detected microbial species in secondary endodontic infections (Tennert et al., 2014). *E. faecalis* is linked to chronic periapical periodontitis and a 50%–70% failure rate in endodontic retreatments (Sakko et al., 2016). Treated root canals provide a well-sealed, low-nutrient environment that enables *E. faecalis* to thrive and exacerbate root canal infections (Alghamdi and Shakir, 2020). The persistence of *E. faecalis* is thought to result from its ability to enter a VBNC state, maintaining viability and pathogenicity through gene expression and metabolic activity. A series of studies have examined the VBNC state in *Enterococcus* spp. under various stress conditions, including starvation, temperature (Heim et al., 2002), light, salinity (Gin and Goh, 2013), and antibiotics (Lleò et al., 2003). Stress factors, such as carbon source exhaustion or incubation in an oligotrophic medium at permissive temperatures, trigger the starvation response (Heim et al., 2002). Activating different metabolic pathways results in changes in the protein profiles of *Enterococcus* Spp. Giard et al. reported starvation-specific alterations in the protein profiles of *E. faecalis*, identifying proteins induced in glucose-starved cells as part of the CcpA regulon. The induction of these enzymes during starvation helps to increase the capacity to scavenge nutrients or mobilize endogenous energetic reserves (Giard et al., 2001). Starved and stationary enterococcal cells can form biofilms on plastic materials, although with reduced efficiency compared to growing cells (Lleo et al., 2007). Sedgley et al. introduced *E. faecalis* into the root canals of extracted single canal teeth and found that *E. faecalis* maintains its viability for 12 months without additional nutrients by entering the VBNC state through cell wall alterations and keeping adhesive properties to cultivated human cells (Sedgley et al., 2005). Signoretto et al. highlighted the structural and biochemical adaptations in VBNC cells, focusing on cell wall changes compared to exponentially growing, stationary, and UV-killed cells (Signoretto et al., 2000). VBNC cells exhibit slight elongation and significantly enhanced mechanical resistance, which is attributed to increased crosslinking in the peptidoglycan structure. Alterations in muropeptide distribution, elevated levels of lipoteichoic acid (LTA), and distinct patterns of penicillin-binding proteins underline the physiological adjustments that facilitate bacterial resilience (Satta et al., 1994). In the VBNC state, peptidoglycan composition shifts significantly, with notable increases in crosslinked muropeptides and elevated activity of autolytic enzymes like muramidase-1, particularly in latent forms (Canepari et al., 1986; Canepari et al., 1987). These findings provide critical insights into bacterial adaptability and persistence, emphasizing the role of the VBNC state in pathogenesis and resistance to environmental and antibiotic stresses, thus contributing to a broader understanding of microbial resilience in challenging conditions. Lieo et al. explored the adhesion and biofilm formation of exponentially growing and nondividing enterococcal cells on polystyrene surfaces under environmental stress using light microscopy. The researchers induced a VBNC state in the exponential phase of enterococcal cells within a microcosm. The cells were exposed to two distinct stress conditions: incubation at 4°C under direct light and at room temperature in the absence of direct illumination. CFU was then assessed every two days until no colonies were observed on solid media, marking the transition to the VBNC state. The viability, biofilm formation, and binding ability of the enterococcal cells in both the microcosm and cultivable suspension were assessed by staining with the LIVE/DEAD kit, adherence assay, and biofilm formation method, respectively. They found that enterococcal cells maintained their adherence and biofilm formation ability under a VBNC state (Lleo et al., 2007). Solid media, even when supplemented with sodium pyruvate, catalase, superoxide dismutase, or reduced agar concentrations, failed to support resuscitation in *Enterococcus* spp. Successful resuscitation was achieved only in liquid media. *E. faecalis* and *E. hirae* exhibited a gradual decline in resuscitable cells, detectable for up to 60 days. In contrast, *E. faecium* showed limited resuscitation, with viable cells detectable for only 7 days. Adding sodium pyruvate, catalase, or superoxide dismutase to liquid media did not improve resuscitation rates (Lleò et al., 2001). These findings underscore significant differences in resuscitation capacity among enterococcal species.

The detection of mRNA, given its inherent instability, is widely used to assess cell viability (Sheridan et al., 1998). Lleò et al. recently demonstrated that the pbp5 mRNA of *E. faecalis* can serve as a marker of VBNC state cell viability, as its presence indicates active gene expression (Lleò et al., 2000; Lleò et al., 2001). To induce the VBNC state, exponentially growing vancomycin-resistant *Enterococcus* cultures were inoculated into oligotrophic microcosms with lake water, some pre-exposed to sub-MIC vancomycin to activate resistance genes, *vanA* and *vanB*, and maintained at 4°C while monitoring CFU counts. This research group further examined the *vanA* and *vanB* genes as markers of the VBNC state (Lleò et al., 2003). The results showed that *E. faecalis* VBNC cells maintained expression of the *vanA* and *vanB* genes for up to one month under the VBNC state. In *E. hirae* and *E. faecalis*, pbp5 mRNA remained detectable for up to 3 months, indicating prolonged viability, while *E. faecium* expressed it for only 2 weeks, suggesting a shorter VBNC state. *E. faecium* showed limited resuscitation capability and rapid cell death, with few cells persisting in the VBNC state. In contrast, *E. faecalis* and *E. hirae* had greater resuscitation potential, sustaining the VBNC state for up to three months. Approximately 1 in 10,000 cells could regain cultivability shortly after entering the VBNC state (Lleò et al., 2000). Another study used SYTO9+PMA-qPCR to assess the viability of *E. faecalis* cells, evaluating cell membrane integrity over 10 days until no cultivable cells were detected (E et al., 2015). The authors used primers targeting the *pbp*5 gene, which encodes a protein involved in peptidoglycan synthesis. Cells in the VBNC state were prepared for TEM and SEM analyses to assess their morphological changes and binding ability to dentin, respectively. Moreover, the acid production ability of the VBNC state cells was monitored every three days. In addition to successfully inducing the VBNC state in *E. faecalis* after 15–30 days, the study revealed significant differences in morphology, glycometabolism, and adhesion properties between VBNC cells and *E. faecalis* in its exponential growth phase. *E. faecalis* could not break down lactose, D-mannitol, or D-sorbitol but metabolized sucrose. Using TEM analysis, the authors observed significant changes in the morphology of VBNC *E. faecalis* cells. The cytoplasmic matrix appeared condensed, and the cells took on an irregular shape, while the cell membranes remained intact. Interestingly, they also found a reduction in *E. faecalis*’s adhesion ability, but not for VBNC *E. faecalis* cells that were still able to adhere to tooth dentine (Postnikova et al., 2015). Sterzenbach et al. developed a LIVE/DEAD qPCR method using modified Propidium Monoazide (PMAxx) to distinguish between viable and nonviable *E. faecalis* in dental hard tissues. PMAxx is a new and improved version of the popular viability dye PMA. Root canals were colonized with *E. faecalis* for three weeks, followed by bacterial inactivation in half of the samples through thermal heating. Samples were treated with varying concentrations of PMAxx, pre-incubated for 30 or 60 min, and cross-linked with DNA using blue light for the same durations. The results confirmed that the untreated viable group showed maximum DNA detection, while PMAxx-treated samples helped assess the bacteria’s viability status by distinguishing live from dead bacterial DNA based on fluorescence and qPCR data. DNA data from ground tooth samples were extracted and analyzed by qPCR to quantify bacterial DNA using a standard curve. qPCR was performed on DNA extracted from tooth samples, with bacterial counts determined from both pellet and supernatant fractions. They recommended this method as useful for evaluating microbial presence in dentin and other hard tissues (Sterzenbach et al., 2024). Therefore, during the VBNC state and low-nutrient sealed environment of root canals, *E. faecalis* maintains its viability and pathogenicity through various biochemical and structural adaptations, including changes in peptidoglycan, lipoteichoic acid levels, and penicillin-binding proteins. Additionally, *E. faecalis* can form biofilms, which further enhance its resilience by protecting bacteria from environmental stresses and antibiotic treatments. This adaptation allows *E. faecalis* to persist in challenging conditions, making it a significant contributor to chronic infections in endodontic therapy.

### 8.3 VBNC state in *H. pylori* and Helicobacter-like organisms (*Campylobacter* spp.)

The oral cavity is considered a potential reservoir for *H. pylori*, which may enter the human body through the mouth and establish lifelong colonization in gastric tissue (Mao et al., 2021). Similarly, positive PCR results could result from *H. pylori* DNA entering the oral cavity via hiccups or contamination through food and water (Mao et al., 2021). Few studies have investigated the VBNC state in oral *H. pylori*, perhaps due to the low prevalence of *H. pylori* in the oral cavity. This low prevalence results from *H. pylori* transient presence in the oral cavity, in addition to the dental plaque microenvironment not supporting its growth. Some studies have detected *H. pylori* in dental plaque and linked its presence to gastroesophageal diseases and poor oral health (Morales-Espinosa et al., 2009; Silva et al., 2010), while others argue that the oral cavity is not a reservoir for the bacterium, with no solid evidence of its isolation (Olivier et al., 2006; Silva-Rossi-Aguiar et al., 2009). Attempts to culture *H. pylori* from the oral cavity have been unsuccessful, raising doubts about its presence and transmission in the mouth (Al-Ahmad et al., 2010). A study investigating the prevalence of *H. pylori* in the oral compartments of infected patients found that despite the high sensitivity of PCR, the bacterium was undetectable in most oral samples, with no correlation to oral health or stomach infection (Al-Ahmad et al., 2012). This suggests that *H. pylori* may only transiently reside in saliva and dental plaque rather than being a permanent resident. Further research has explored how *H. pylori* survives in human saliva and how oral microorganisms influence its survival (Scholz et al., 2025). Two *H. pylori* strains, were cultured in pooled human saliva or Brucella broth formula (BBF), either alone or with oral microorganisms like *Streptococcus mutans*, *Lacticaseibacillus casei*, *Streptococcus oralis*, *Actinomyces naeslundii*, and *Candida dubliniensis*. The two strains were KE 88-3887, a motile derivative of *H. pylori* 26695 and SS1 (*H. pylori* Sidney Strain 1), which was originally isolated from a gastric mucosa biopsy of an *H. pylori-*positive patient (Lee et al., 1997). The results showed that *H. pylori* KE 88-3887 survived longer in co-culture with as *S. mutans* and *A. naeslundii*, but could not be cultured after 168 hours. *H. pylori* SS1 remained viable after 168 hours when co-cultured with *S. mutans* and *C. dubliniensis*, but not in mono-culture. These findings suggest that *H. pylori* can transiently survive in human saliva with certain oral microorganisms but is not a permanent member of the oral microbiota. All in all, the literature on *H. pylori* to date describes the detection of this bacterium using molecular biological or biochemical methods. The isolation of oral strains of *H. pylori* has not been described in the literature to date, as described in a detailed review published by our group (Mao et al., 2021). In this aforementioned review, it became clear that the previous detection of *H. pylori* in the oral cavity is more likely due to the fact that it is transient in the oral cavity. However, one reason for the previous absence of oral *H. pylori* strains could be the conversion of this germ into VBNC in the oral cavity, as discussed in this review.

Site-specific factors, such as pH value and nutrient availability, may influence the ability of *H. pylori* to survive in the mouth (Oshowo et al., 1998). Since *H. pylori* typically resides in the gastric mucosa, the bacterium is likely to prefer mucosal epithelial areas within the mouth over dental plaque. Furthermore, certain bacterial species in the oral cavity may inhibit *H. pylori* growth. However, *H. pylori* exhibits selective adherence to *Fusobacterium* spp. as an important bridging microorganism connecting non-coaggregating bacteria such as *H. pylori* in dental plaque and their establishment within the plaque matrix (Andersen et al., 1998). Few studies have demonstrated the connection between periodontitis-associated bacteria and *H. pylori* presence in the oral cavity (Hu et al., 2016). Studies have indicated that *P. gingivalis*, *T. denticola*, and *P. intermedia* are more prevalent in *H. pylori*-positive individuals than in *H. pylori*-negative ones (Kadota et al., 2020). A recent study suggests that *H. pylori* elimination from the oral cavity is crucial to prevent its colonization in the stomach, particularly in patients with periodontitis (Umeda et al., 2003). Dental plaque biofilms shield *H. pylori* from systemic antimicrobial treatments, rendering it resistant to such therapies (Gebara et al., 2006). Researchers suggest that *H. pylori* recolonizing the gastric mucosa from dental plaque remains unaffected by synthetic antimicrobials. Thus, periodontal therapies targeting microbial deposits, including *H. pylori*, within dental plaques are essential for effective management (López-Valverde et al., 2022). *H. pylori* can adapt to harsh oral conditions in response to synergistic interactions with oral microorganisms by transitioning to a VBNC or dormant state. An example of this is its symbiotic relationship with Candida, where fragments of *H. pylori* genes are found in the DNA of oral yeasts. This suggests that *Candida* aids in re-inoculating *H. pylori* in the stomach or transmitting it to new hosts (Costa et al., 2024). A study detected *Streptococcus mitis* in human gastric biopsies and showed that it induced *H. pylori* to transform into a VBNC coccoid form while enhancing its own cultivability in coculture (Khosravi et al., 2014). Another study extended the analysis by examining the varying protein profiles of *H. pylori* and *S. mitis* in a multispecies environment. In cocultivated *H. pylori*, proteins associated with RNA degradation, DNA repair mechanisms, and LPS biosynthesis increased, while those involved in energy production, translation, metabolism, and cell signaling decreased. While the coccoid transformation of *H. pylori* is expected, the increased survival of *S. mitis* suggests a potential pathogenic role that warrants further investigation in the gastric environment (Khosravi et al., 2016). Coccoid *H. pylori* exhibits reduced metabolic enzyme levels but maintains high levels of DNA biosynthesis proteins. Although it cannot be cultivated *in vitro*, the coccoid form shows stronger antiproliferative and weaker proapoptotic effects compared to its spiral form, suggesting a potential role in gastric cancer progression (Li et al., 2013).

**Figure 3:**
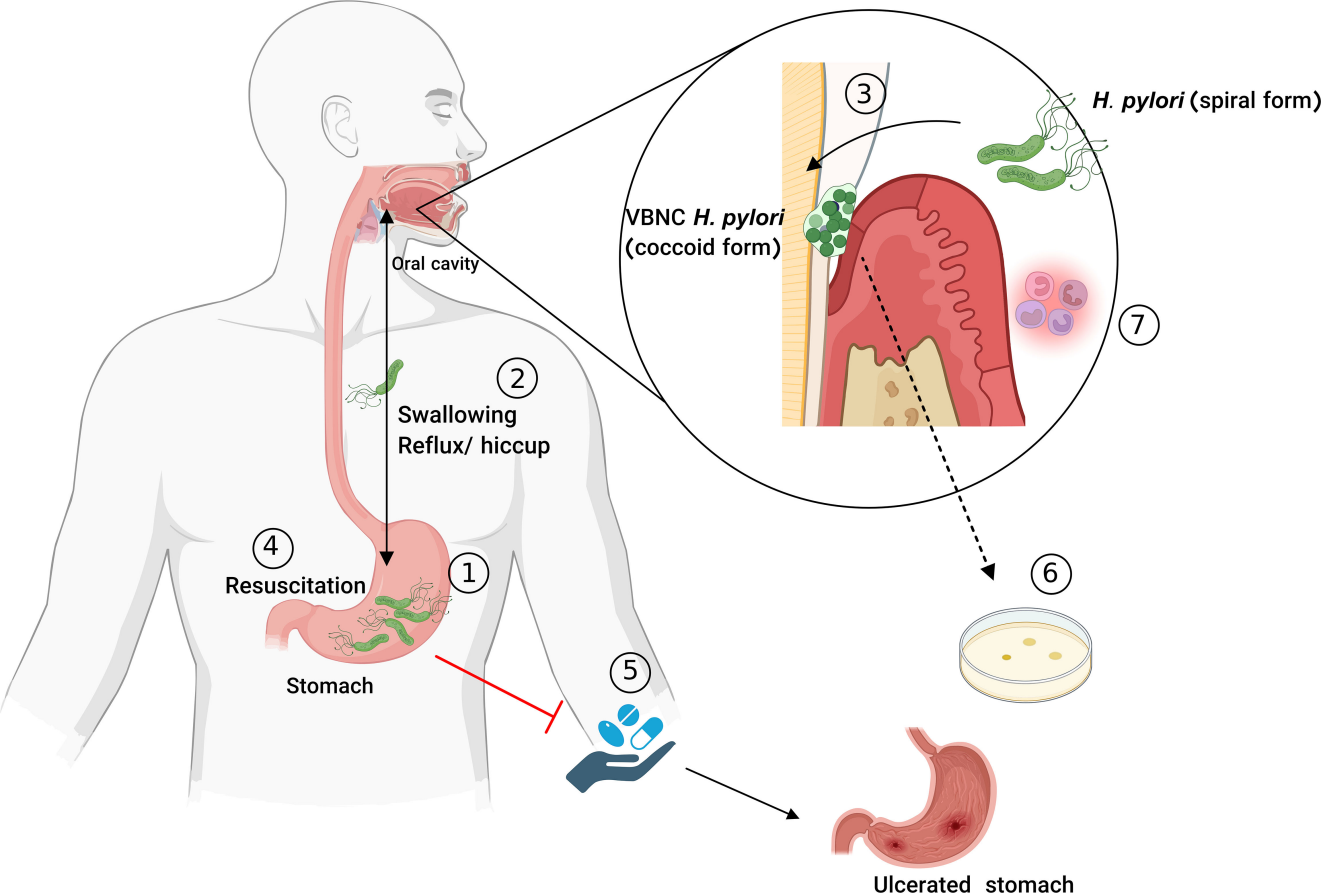
The oral cavity as a potential source of *H. pylori* gastric reinfection. *H. pylori* can transiently transfer from the stomach (1) to the oral cavity during reflux events (2) and persist in dental plaque, caries, and periodontal pockets by transitioning into a coccoid VBNC state to adapt to adverse oral conditions (3). Upon swallowing, *H. pylori* can resuscitate in the stomach (4), contributing to reinfection, therapy failures (5), and chronic gastric diseases. The VBNC state is difficult to cultivate (6), and *H. pylori* synergistically interacts with oral microbes, exacerbating inflammation and potentially contributing to periodontitis (7). (*H. pylori*: *Helicobacter pylori*, VBNC: viable but non-cultivable).

Eradicating *H. pylori* effectively reduces gastric cancer risk, but recurrence remains a global concern, particularly in underdeveloped regions with a high prevalence. Recurrence within a year is often classified as relapse, while reinfection typically occurs later, driven by socioeconomic and environmental factors (Zhang et al., 2024). Recurrence rates vary widely, ranging from 0.2%–6.2% in developed countries to 2.2%–73% in developing regions (Yan et al., 2013; Hu et al., 2017). A 2024 study by Zhang et al. reported a 1-year recurrence rate of 3.2%, attributed to poor sanitation and regional differences (Zhang et al., 2024). Addressing these disparities is essential to mitigating *H. pylori*-associated risks globally. Recurrence and reinfection are key processes by which *H. pylori* reemerges after successful eradication, with patients potentially reinfected with either the same strain or a different one (Adachi et al., 2002). Ahuja-Vineet et al. demonstrated that the recurrence rate is significantly higher with imidazole-based treatments, such as nitroimidazole and ranitidine bismuth citrate, compared to non-imidazole regimens, such as furazolidone (Salcedo and Al-Kawas, 1998; Hildebrand et al., 2001). Low-efficacy therapy regimens often temporarily reduce *H. pylori* levels in the gastric mucosa, thus contributing to recurrence. The VBNC state of *H. pylori* is closely linked to treatment failures, recurrent infections, and poor clinical outcomes (Salcedo and Al-Kawas, 1998; Ahuja and Sharma, 2002). Oral *H. pylori* may colonize the oral cavity as a non-cultivable form if supragingival plaque contains low levels of AI-2 (Krzyżek and Gościniak, 2018), so identifying *H. pylori* in the oral cavity as a potential reservoir could provide an effective strategy for its complete eradication (Zhang et al., 2022). *H. pylori* can transition into a VBNC state when exposed to stressors, such as anaerobic environments, nutrient deprivation, or prolonged liquid culture, adopting a spherical shape and losing cultivability (Hirukawa et al., 2018). While its virulence decreases compared to its helical form, VBNC *H. pylori* retains pathogenicity, as shown by its ability to colonize mouse gastric walls and induce mucosal damage (Cellini et al., 1994). As shown in **Figure 3**, it may temporarily transfer from the stomach to the oral cavity during gastroesophageal reflux or hiccups, but the oral cavity’s unfavorable conditions, such as unstable temperatures, high oxygen levels, and microbial competition, promote the transition to the VBNC state. This transition involves physiological and morphological changes, complicating viability assessments in antibacterial studies. In biofilms, factors like temperature fluctuations, low nutrients (Bowen et al., 2018), acidity, hypoxia (Krzyżek et al., 2020), and interactions with other oral bacteria further enhance *H. pylori*’s survival and resistance, allowing it to persist in dental plaque biofilms, carious cavities, and periodontal pockets. VBNC state-like coccoid forms of *H. pylori* have been identified in oral samples (Hirsch et al., 2012; Cieplik et al., 2019), suggesting enhanced resistance to harsh conditions (Zhang et al., 2022). When swallowed and re-entering the stomach, *H. pylori* can resuscitate, leading to reinfection, therapy failures, and the development of chronic ulcers and gastritis. The VBNC state poses substantial clinical challenges because these forms cannot be cultivated using conventional microbiological methods. Furthermore, *H. pylori* can adhere to and invade oral cells, interacting synergistically with other oral microorganisms to exacerbate inflammation and potentially contribute to periodontitis.

Hirukawa et al. studied the transition of *H. pylori* from the microaerophilic stomach into the anaerobic intestinal environment, which resembles the gingival pocket and dental plaque environment (Hirukawa et al., 2018). The authors examined the morphological changes induced by anaerobiosis in *H. pylori* using SEM and differentiated the results in all forms of *H. pylori* using the western blot technique. Urease activity remained in all forms of *H. pylori*, but a loss of motility and the cytotoxin-associated antigen (cagA) secretion system were observed during its transition to the VBNC state (Hirukawa et al., 2018). The long chromosomal region in *H. pylori* known as cagPAI codes the type IV secretion system (T4SS) and the 125–145 kDa effector protein CagA. T4SS facilitates the translocation of CagA into gastric epithelial cells (Tegtmeyer et al., 2011). *H. pylori* types that carry cagPAI and express CagA are extremely virulent, while cagPAI negative strains of *H. pylori* are less virulent. Some illnesses, including chronic gastritis, peptic ulcer diseases, and stomach cancer, have been linked to *H. pylori* strains that produce CagA (Ahmadzadeh et al., 2015).

Since *H. pylori* has not yet been cultivated in the oral cavity, reliable detection requires a combination of molecular, biochemical, and immunological assays to reduce false-positive results in this environment (Mao et al., 2021). One of the most difficult issues with *H. pylori* is its ability to transform from a spiral to a coccoid form in response to environmental stressors or antibiotics, during which the bacteria become unable to revive the coccoid form of infection (Cellini et al., 1994). Amoxicillin effectively inhibits the spiral form of *H. pylori*, while a 2x MIC of amoxicillin has no bactericidal effect on the coccoid form. SpoT, the main transcriptional regulator, plays a crucial role not only in the persistence of *H. pylori* but also in its ability to evade phagocytosis by macrophages. Similar to other VBNC bacteria, *H. pylori* shows no capacity for multiplication and minimal metabolic activity, raising the possibility of drug-resistant and recurrent infections (Ozcakir, 2007). The VBNC state of *H. pylori* was studied using ½ MIC of amoxicillin (after 144 h), and then confirmed by Gram-staining and flow cytometry using propidium iodide. A lower rate of *cagE* and *babA* (Poursina et al., 2013), as well as an increase in *spoT* (Poursina et al., 2018), was observed in the non-cultivable coccoid form of *H. pylori* compared to the spiral. Therefore, VBNC state *H. pylori* may induce chronic infection through the (low but still) activity of *cagE* and *babA* and to confer antibiotic resistance through high-level expression of *spoT*. Young et al. analyzed the morphological changes of *H. pylori* in gastric biopsies and supragingival dental plaque using SEM (Young et al., 2001). The authors prepared beads comprising bacterial aggregates and studied them using SEM and PCR for the urease gene. However, their study was the first to visualize *H. pylori* in dental plaque through SEM and revealed no morphological differences in *H. pylori* cells between gastric biopsy and dental plaque samples (Young et al., 2001). The association between the coccoid form of *H. pylori* and its VBNC state seems to be a controversial subject because the coccoid forms of *H. pylori* are categorized into three types based on their morphology: the degenerative coccoid form (dying organisms), viable coccoid bacteria capable of cultivation in agar medium; and the VBNC state of *H. pylori* incapable of growth on agar (Saito et al., 2003). In this situation, metabolic activity assessment using flow cytometry may be a good method for differentiating among these different coccoid forms. The metabolic rate of coccoid forms is notably reduced when compared to spiral forms, but it is not completely stopped. To assess protein synthesis in the VBNC state of *H. pylori*, Loke et al.’s findings through Sequential Window Acquisition of all Theoretical Mass Spectra (SWATH-MS) showed that most of the proteins involved in DNA replication, cell division, and biosynthesis are still produced in the VBNC state (Loke et al., 2016; Zhao et al., 2016).

*Campylobacter* spp. are extremely difficult to cultivate and can enter a VBNC state under stress conditions, such as low temperatures, oxygen exposure, and nutrient deprivation. This state has been observed in both environmental and clinical strains, with its occurrence being strain dependent. Notably, the VBNC state may reverse after passage through a host, as demonstrated in strains isolated from broiler house soil (Santos et al., 2023). A *C. jejuni* strain required 38 days to transition into the VBNC state, highlighting the need for further research on its prolonged viability and associated food safety risks. This transition involves morphological and metabolic adaptations, including modifications in the cell wall, membrane, and capsule structure, which enhance survival under adverse conditions (Chaisowwong et al., 2012). Notably, VBNC *C. jejuni* hyper-expresses 2,4-di-tert-butylphenol, an antioxidant phenol compound that mitigates DNA damage from oxidative stress caused by agents like hydrogen peroxide, thereby supporting cell viability (Santos et al., 2023). Beyond *C. jejuni*, the ability to enter the VBNC state has also been documented in *C. hepaticus*, *C. coli*, and *C. lari* (Dong et al., 2019; Lv et al., 2020; Phung et al., 2022)—findings for *C. hepaticus* were discussed earlier in the nonmedical context of VBNC. The ability of *Campylobacter concisus* to enter the VBNC state was examined through a 3-week incubation at 4°C. Wahid et al. detected the viability of *C. concisus* using PMAxx-qPCR (DNA gyrase subunit B). The VBNC state lasted for 9–15 days, and analysis of cell morphology through viability assay and TEM showed a conversion to smaller coccid cells (Wahid et al., 2024).

Although further genomic and proteomic studies are needed to clarify VBNC state resuscitation mechanisms, factors promoting the resuscitation of *C. jejuni*, *C. coli*, *C. hepaticus*, and *C. concisus* VBNC cells include animal models, enriched media with supplements such as ferrous sulfate, sodium metabisulfite, sodium pyruvate, and ʟ-cysteine (Chou et al., 1983; Phung et al., 2022), as well as temperature upshifting and modified *Campylobacter*-selective agar (Vilhena et al., 2019). Pyruvate aids resuscitation by scavenging ROS, preventing lipid peroxidation (Pan and Ren, 2022), and stimulating DNA and protein biosynthesis (Zhang et al., 2021). In their study, significant morphological changes were observed between resuscitated cells, cells in the exponential growth phase and campylobacter in the VBNC state using LM, CLSM, and TEM. Resuscitated and exponential-phase cells displayed elongated rod or arc shapes, while VBNC *Campylobacter* species transformed into short rods or cocci with irregular and distorted morphologies (Wahid et al., 2024).

In summary, the oral cavity serves as a potential reservoir for *H. pylori* and *Campylobacter* spp., where they can survive by forming a supportive microenvironment, such as dental plaque. The ability of these bacteria to enter a VBNC state allows them to persist in the oral cavity despite the lack of ideal growth conditions. This state enables *H. pylori* to evade detection and survive environmental stresses, including the use of antibiotics, which markedly contribute to the challenges of complete eradication. Moreover, the VBNC state of *H. pylori* is associated with recurrent infections and poor clinical outcomes, as it maintains minimal metabolic activity but retains the potential for chronic infection and antibiotic resistance. Identifying *H. pylori* in the oral cavity may offer critical insights into its persistence and pathogenesis, providing an avenue for more effective strategies for eradication.

## 9 Conclusions and future research directions

The VBNC state is widely recognized as a defense mechanism that enables bacteria to withstand various stressors, including pasteurization, antibiotics, and oxidation. Deeper insights into the VBNC state of bacteria and the underlying mechanisms are crucial for managing the environment, food safety, agricultural productivity, and healthcare settings. Although studies are abundant in this regard, further investigations are still required to understand the precise mechanism of the formation and resuscitation of VBNC cells in general. This is because the mixed system used in the studies conducted so far, which includes dead, cultivable, and damaged cells, causes interferences, resulting in many gaps in knowledge. As a result, future studies should prioritize separating VBNC cells from mixture systems. While several detection techniques have been established, each has its own advantages and drawbacks, necessitating the development of new techniques that are quick, sensitive, affordable, and simple to use to identify VBNC cells. Given the advantages and drawbacks of each technique, employing multiple methods is often necessary rather than relying on a single approach. Determining the proper and optimum dosage of dye concentrations, such as PMA, is essential for accurate viable cell quantification and to prevent false-negative results. Utilizing excessive or low concentrations of PMA can lead to estimation errors by either inhibiting DNA amplification in living cells or failing to adequately suppress the signal from dead cells. The VBNC state has been reported in only a few oral bacteria, despite the oral biofilm containing more than 700 different bacterial species. Therefore, future studies should intensively search for the VBNC state in the oral cavity. This is particularly important because antimicrobial mouthwashes, such as those containing chlorhexidine digluconate and cetylpyridinium chloride, are commonly used and can serve as key stress factors for oral microorganisms. Particular attention should be given to *H. pylori*, which may exist in the oral cavity in a transient active but nongrowing form, potentially playing a crucial role in its infection cycle.
